# An In-Depth Review on Sensing, Heat-Transfer Dynamics, and Predictive Modeling for Aircraft Wheel and Brake Systems

**DOI:** 10.3390/s26030921

**Published:** 2026-01-31

**Authors:** Lusitha S. Ramachandra, Ian K. Jennions, Nicolas P. Avdelidis

**Affiliations:** 1 IVHM Centre, Faculty of Engineering and Applied Sciences, Cranfield University, Bedford MK43 0AL, UK; i.jennions@cranfield.ac.uk; 2 Department of Aeronautics and Astronautics, School of Engineering, University of Southampton, Southampton SO17 1BJ, UK; n.p.avdelidis@soton.ac.uk

**Keywords:** aircraft wheels and brakes, condition monitoring, heat transfer dynamics, machine learning prediction models, physics-informed and hybrid modeling, brake temperature prediction

## Abstract

An accurate prediction of aircraft wheel and brake (W&B) temperatures is increasingly important for ensuring landing gear safety, supporting turnaround decision-making, and allowing for more effective condition monitoring. Although the thermal behavior of brake assemblies has been studied through component-level testing, analytical formulations, and numerical simulation, current understandings remain fragmented and limited in operational relevance. This paper discusses research across landing gear sensing, thermal modeling, and data-driven prediction to evaluate the state of knowledge supporting a non-intrusive, temperature-centric monitoring framework. Methods surveyed include optical, electromagnetic, acoustic, and infrared sensing techniques as well as traditional machine-learning methods, sequence-based models, and emerging hybrid physics–data approaches. The review synthesizes findings on conduction, convection, and radiation pathways; phase-dependent cooling behavior during landing roll, taxi, and wheel-well retraction; and the capabilities and limitations of existing numerical and empirical models. This study highlights four core gaps: the scarcity of real-flight thermal datasets, insufficient multi-physics integration, limited use of infrared thermography for spatial temperature mapping, and the absence of advanced predictive models for transient brake temperature evolution. Opportunities arise from emissivity-aware infrared thermography, multi-modal dataset development, and machine learning models capable of capturing transient thermal dynamics, while notable challenges relate to measurement uncertainty, environmental sensitivity, model generalization, and deployment constraints. Overall, this review establishes a coherent foundation for thermography-enabled temperature prediction framework for aircraft wheels and brakes.

## 1. Introduction

Aircraft has greatly improved global connectivity by facilitating rapid, long-distance transportation and trade, integrating international markets and industries, and enabling faster emergency responses. This level of capability has been made possible by the use of modern aircraft, which benefit from highly advanced operational practices, well-established maintenance procedures, and exceptional component reliability. These factors work in harmony to ensure consistent safety, efficiency, and dependability across global air transport networks. As with other key aircraft subsystems, the reliability and availability of the Landing Gear wheels and brakes (W&B) are essential for ensuring safe operations. Routine (planned) maintenance is crucial to maintain their performance, enabling smooth landings, effective deceleration, and safe ground handling while reducing the risk of failures during critical flight phases. Although routine maintenance is carried out in airline operation, operational interruptions may occur due to technical defects. Unscheduled maintenance interventions can impose substantial costs on airlines. Therefore, minimizing unscheduled maintenance and optimizing the operational schedule are of paramount importance. For the aircraft wheels and brakes (W&B) subsystem, one effective approach to cost reduction is the development of a condition monitoring and prediction system based on non-invasive methodologies. Such approaches enable the capture of key parameters without the need for additional onboard sensors on aircraft, thereby avoiding the need for further certification or system modifications. Thermography is a non-invasive technique capable of capturing temperature distributions across W&B subcomponents without physical contact. Implementing a temperature-based condition monitoring and prediction system for W&B could significantly reduce maintenance costs, enhance schedule planning and management, improve safety, lower labor hours, reduce aircraft ground time, and ultimately increase aircraft dispatch reliability.

**Figure 1:**
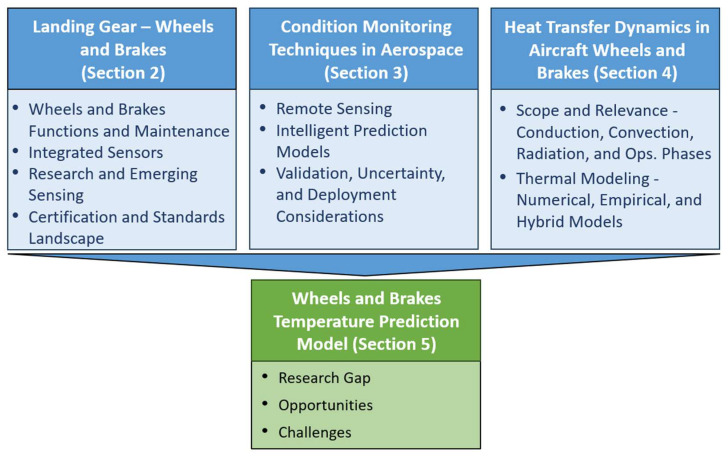
The thematic scope of the literature review.

In order to support the development of the proposed approach, a comprehensive literature review was conducted to critically assess prior work within the relevant research domains. This review article has been compiled to provide an integrated overview of the key areas that must be understood and further research pursued before advancing toward the development of ‘a condition monitoring and prediction framework’ for aircraft landing gear wheels and brakes. The thematic scope of this review is outlined under four different areas, depicted in Figure 1. Section 2 of this review discusses aircraft landing gear with particular emphasis on wheels and brakes. It outlines their primary functions, current maintenance practices, integrated sensing technologies, ongoing research, emerging sensing approaches, and the certification and standards framework relevant to wheel and brake sensors. Section 3 then explores condition monitoring within the aerospace domain, covering established techniques, remote sensing methods, and intelligent prediction models with a focus on machine learning. Section 4 addresses the heat-transfer dynamics of wheels and brakes, including conduction, convection, and radiation pathways, as well as thermal modeling approaches—numerical, empirical, and hybrid. To conclude this background survey, Section 5 synthesizes the key research gaps, potential opportunities, and persisting challenges identified across existing literature, with a particular focus on the development of a robust condition monitoring and temperature predictive framework for aircraft landing gear wheels and brakes.

The literature reviewed in this paper was established through structured searches of major scientific databases, including Scopus, IEEE Xplore, Web of Science, and ScienceDirect. Search terms combined application-specific keywords with cross-disciplinary concepts related to heat transfer, thermodynamics, materials behavior, and machine learning. Priority was given to peer reviewed journal articles, conference papers, and relevant standards addressing sensing technologies, heat-transfer mechanisms, and predictive modeling. While most of the sources reside in the aerospace engineering domain, foundational studies from thermodynamics, materials science, and data-driven modeling were incorporated where they directly supported the understanding of wheel and brake thermal behavior and prediction. This approach reflects the application-driven focus of the review while maintaining cross-disciplinary integration.

## 2. Landing Gear Wheels and Brakes

The evolution of wheeled landing gear systems began shortly after the Wright Brothers’ pioneering flight in 1903. By 1906, Santos-Dumont’s “No. 14 bis” had introduced one of the earliest documented applications of wheeled gear in Europe [1]. This innovation was rapidly adopted in subsequent aircraft developed between 1907 and 1909 by aviation pioneers such as Voisin, Farman, Bleriot, Curtiss, and others—several of whom achieved milestone flights across various regions [2]. By the onset of World War I, landing gear configurations had largely converged toward tailwheel designs, featuring robust strut assemblies and primitive shock absorption mechanisms [3]. As the aircraft landing gears are carried for most of the mission but used only during taxi, take-off, and landing phases, it should be engineered to the lowest practicable mass consistent with safety. In practice, designers size the gears to meet all certified worst-case load scenarios and thermal requirements (e.g., landing impact, rejected take-off brake energy, taxi/ground-handling, fatigue) and then minimize structure and actuation mass via efficient architectures and high specific-strength materials that are durable [4]. This marked the foundation of modern landing gear systems, setting the stage for continuous advancements in structural resilience, energy dissipation, and operational reliability.

### 2.1. Wheels and Brakes Functions

The landing gear system plays a critical role in ensuring the safe ground operation of an aircraft by enabling controlled take-off, taxiing, and landing. Its primary function is to absorb and dissipate the substantial impact forces and moments generated during landing and ground maneuvers, transferring these loads safely into the airframe. In addition to this core responsibility, landing gear supports several essential secondary functions. It facilitates ground maneuverability through steering during taxiing and take-off, enables braking after touchdown or during low-speed movements, and in the case of large aircraft, accommodates towing and pushback by airport vehicles. Furthermore, it ensures proper load distribution to prevent damage to airport surfaces by optimizing the number and configuration of wheels [5]. The landing gear system is essential for safe ground operations, serving both primary functions such as absorbing landing impacts and secondary roles, including steering, braking, towing, and load distribution, all of which are crucial for efficient and damage-free aircraft operation on the ground.

**Figure 2:**
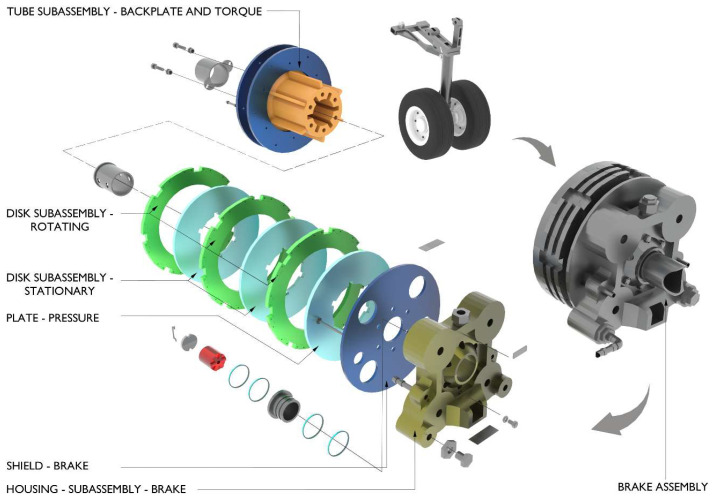
An illustration of a typical commercial aircraft wheel brake unit. Diagram based on reference [6].

Integral to the landing gear system, wheels and brakes play a vital role in aircraft deceleration and ground handling, particularly during landing roll and aborted take-off scenarios. Modern aircraft predominantly utilize carbon–carbon composite brakes, which offer superior thermal performance and mechanical efficiency compared to traditional steel alternatives. An exploded view of a typical carbon–carbon rotor–stator arrangement in a commercial aircraft brake unit is illustrated in Figure 2 [6]. The figure highlights the principal subassemblies of the wheel and brake unit, including the torque tube and backplate, alternating rotating and stationary carbon discs forming the friction stack, the pressure plate, and the brake housing with its associated actuation components. The rotating discs are mechanically coupled to the wheel, while the stationary discs are splined to the torque tube to transmit braking torque into the landing gear structure. The brake shield is positioned to limit radiative heat transfer toward the wheel and tire. The curved arrows in Figure 2 indicate the transition from the fully assembled wheel and brake installed on the landing gear to the exploded view configuration and do not represent operational motion. These systems operate by converting aircraft’s kinetic energy into thermal energy through friction generated between stator and rotor discs, actuated by hydraulic or electric systems that ensure controlled braking without wheel lock-up. Carbon brakes are notably lighter, contributing to reduced overall aircraft weight, improved fuel efficiency, and enhanced payload capacity. Their high thermal stability allows for effective operation under extreme temperatures, significantly reducing the risk of ‘brake fade’ during high-intensity braking [7,8]. Furthermore, their resistance to wear and environmental degradation results in lower maintenance frequency and cost over the aircraft’s lifecycle [5]. However, while carbon brakes demonstrate clear operational and economic advantages, their long-term performance should be re-evaluated in the context of evolving aircraft specifications such as higher certified landing weights, reduced turnaround intervals, and integration with more-electric braking architectures to ensure their design capabilities and material properties remain suited to future aircraft platforms.

### 2.2. Wheel and Brake Maintenance

In routine operations, landing gear, wheels, and brakes are subject to repeated stress and degradation during taxi, take-off, and landing phases. Their reliability is essential for flight safety, and as such, these components require regular monitoring. Maintenance demand for these systems is closely tied to flight cycle (FC) utilization, making it a key driver of their service intervals. These systems are categorized under Air Transport Association Chapter 32, which outlines the components and functions essential for the operation of landing gear systems [9]. Although grouped together in maintenance, with repair and overhaul (MRO) programs as a distinct airframe maintenance category, the individual components of landing gear systems do not necessarily share aligned removal or servicing intervals. Some maintenance actions are easy to schedule based on predefined usage thresholds—such as a set number of FC, flight hours, or years, whichever occurs first. This enables predictable maintenance planning, allowing airlines to coordinate landing gear servicing with routine base checks or plan in-service gear swaps, reducing aircraft downtime.

From a cost-management perspective, most landing gear maintenance is outsourced to original equipment manufacturers or specialist MRO providers through fixed-overhaul agreements. These typically include routine work-scopes such as labor, tooling, and standard consumables. However, additional tasks—such as non-routine work, compliance with service bulletins, airworthiness directives, and unexpected part replacements—may fall outside these agreements, introducing financial and logistical complexities. While some elements of landing gear and brake maintenance are highly predictable, others are more reactive in nature, requiring flexible planning and robust operational oversight. Routine inspection and proactive maintenance of aircraft wheel and brake systems are essential to minimize unscheduled shop visits, particularly for high-utilization operators such as low-cost carriers. Visual inspections, however, vary in execution due to subjective interpretation by line mechanics, which can impact the consistency of defect detection. While maintenance manuals provide clear criteria for repairs and removals, inspection protocols remain loosely defined, leaving airlines to integrate their own inspection routines within their Aircraft Maintenance Program. As such, inspection frequency and thoroughness can differ significantly across operators and regions [10]. While landing gear, wheels, and brakes fall under a common Air Transport Association Chapter 32 framework and allow for predictable maintenance scheduling in some cases, differences in wear patterns, inspection practices, and contractual maintenance arrangements introduce variability and complexity, requiring both structured planning and adaptable oversight to ensure safety, operational efficiency, and cost control.

Brake systems on commercial aircraft typically consist of three main elements: the piston housing, heat sink (with alternating rotors and stators), and the torque plate. These systems are wear-monitored using visual indicators such as wear pins. About 95% of brake removals are due to brake wear identified via these indicators. Removal intervals vary widely depending on brake type and usage. Steel brakes, typically installed on aircraft such as the Boeing 757 and 767, require shop visits (SVs) after 1200 to 1800 FC, whereas newer carbon brakes used on the A320, A330, and 777 can last between 2000 and 3000 FC. Although carbon brakes are more expensive to purchase and repair, they offer lower cost-per-cycle, faster overhauls, and weight savings—which can benefit fuel efficiency depending on the airline’s flight profile [11]. Advancements in brake technology, particularly electronically actuated carbon brakes, have introduced wear-pin sensors that estimate the remaining thickness of brake pads. These sensors, embedded in each brake pad, provide a physical measurement that decreases as the pad material wears. Wear-pin measurements are often recorded intermittently—typically once every ten flights—and serve as the primary signal for assessing brake wear. An analysis of wear-pin measurements enables the estimation of brake degradation on a per-flight basis and the classification of wear severity into categories such as high, medium, or low [12]. This underscores the shift toward data-driven monitoring and predictive analytics in brake maintenance, enabling operators to optimize replacement intervals, reduce unscheduled removals, and enhance overall operational efficiency.

Wheel assemblies include multiple components—tires, bolts, bearings, heat shields, and hubs—all of which must be dismantled and inspected during tire-change shop visits. The benefits of regular inspection, noting that approximately 80% of one client’s fleet incurs no additional cost during SVs due to consistent oversight. In contrast, operators relying solely on overhaul SVs frequently face unexpected costs due to neglected part wear or damage. Randell reports that 100% of overhaul-only customers require part replacements, with around 25% facing extra expenses due to specialist repairs. Tire condition typically determines wheel removal intervals. Since wheel maintenance is on-condition, actual SVs are often triggered by observable issues such as wear, cuts, pressure loss, rejected take-offs, or foreign object damage. Environmental and operational factors like temperature and runway conditions also influence component wear. For example, summer heat accelerates tire wear, and under-inflated tires can lead to premature shoulder damage. Tread life for main wheels generally ranges from 175 to 250 FC, while nose wheel treads last between 250 and 350 FC [11]. These insights highlight that while brake and wheel system longevity is influenced by component design and material choice, optimal service life and cost efficiency ultimately depend on proactive inspection regimes, operational conditions, and strategic maintenance planning tailored to each operator’s fleet profile.

According to a 10 year MRO spend forecast by ICF (in constant 2016 USD), shown in Table 1, global maintenance expenditure for landing gear, wheels, and brakes varies significantly by aircraft type, reflecting both fleet trends and operational profiles. The data show continued growth in MRO spending for the 737, A320, and 777 fleets, particularly in landing gear maintenance, where the 737 segment is expected to see a compound annual growth rate (CAGR) of 6.3%, reaching over USD 233 million by 2026. Similarly, the A320 family shows strong growth in both landing gear (5.7% CAGR) and wheels and brakes (4.4% CAGR), with spend projected to exceed USD 1.98 billion in wheels and brakes alone. Conversely, legacy aircraft such as the 757, 767, and A330/A340 show declining MRO demand across both components. For example, wheel and brake MRO for the 757 is expected to decline sharply with a CAGR of −12.5% and the 767 by −7.2%, reflecting the ongoing retirement and reduced utilization of these fleets. These trends underscore a shift in global fleet composition toward newer, high-utilization narrowbodies like the 737 and A320 families, which are increasingly dominating both operations and maintenance investment. Notably, the “Others” category—which likely includes newer widebody and regional jet platforms—is projected to grow steadily, indicating rising MRO demand for emerging or less conventional fleets [11]. Overall, the forecast illustrates how evolving fleet demographics and utilization patterns are reshaping global MRO demand, concentrating investment on high-cycle, fuel-efficient aircraft families while progressively diminishing the maintenance footprint of aging, less economically viable platforms.

$$CAGR={\left(\frac{Ending\;Value}{Beginning\;Value}\right)}^{\frac{1}{n}}-1$$

where

- *CAGR* is the compound annual growth rate;
- *Ending Value* is the value at the end of the period (e.g., 2026 MRO spend);
- *Beginning Value* is the value at the start of the period (e.g., 2016 MRO spend);
- *n* is the number of years (in this case, 10 years).

#### 2.2.1. Integrated Sensors on Wheels and Brakes

In present-day transport aircraft, a dedicated suite of sensors is integrated within the landing gear wheels and brake assemblies to fulfill three main purposes: (i) enable vital control systems such as antiskid and autobrake, (ii) protect hardware through monitoring of overheating and wear, and (iii) support maintenance planning and enhance dispatch reliability. Although specific architectures vary across airframers and suppliers, common implementations include wheel-speed transducers, which are mounted in the axle to feed the Brake and Steering Control Unit (BSCU) for slip control [13]; brake-temperature probes are embedded in the brake structure to detect hot brakes; tire-pressure transducers transmit the pressure signals via wires or wireless systems such as Smart-Stem systems that replace valve stems and transmit to a Tire and Brake Monitoring Unit (TBMU) [14]; and in electric brake systems, embedded sensors for position, load, and wear provide local health monitoring [15]. In modern aircraft, the data acquired from these devices are typically processed by a TBMU or BSCU, subsequently relayed to cockpit display systems such as Engine Indicating and Crew Alerting System (EICAS) or Electronic Centralized Aircraft Monitor (ECAM) and archived for maintenance diagnostics and record-keeping [16].

##### Wheel-Speed Sensors

Wheel-speed sensing is widely employed across aircraft wheels, most prominently to support antiskid (anti-lock) brake control. In such applications, wheel-speed transducers monitor the time-varying rotational kinematics (angular position and/or velocity) of the wheel and deliver a corresponding signal—analog or digital—to the brake-control logic and associated monitoring functions [17]. Two general configurations of wheel-speed sensors are in operational use: (i) alternating-current (AC) and (ii) direct-current (DC) systems, which are functionally equivalent except for the design of the wheel-speed sensors and a corresponding variation in the control unit circuitry.

**Figure 3:**
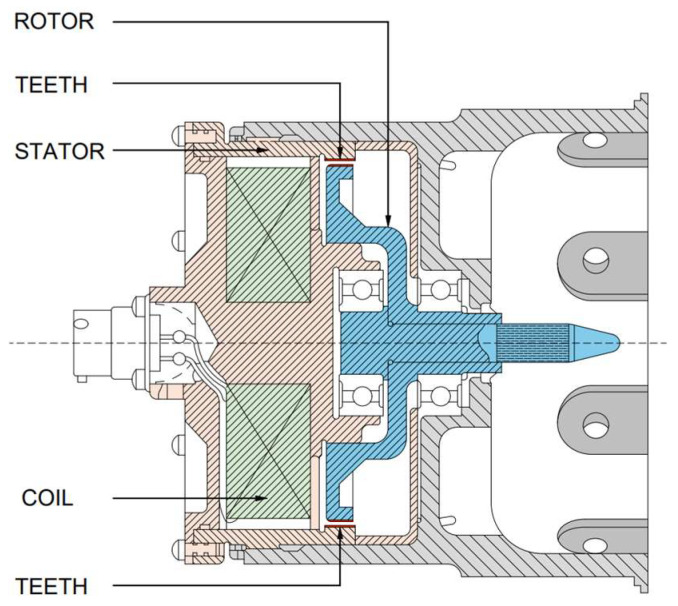
The internal arrangement of an AC wheel-speed transducer. Diagram based on reference [18].

In AC-based configurations, the wheel-speed sensor operates as a variable-reluctance AC generator. This device incorporates a permanent magnet encased by a pickup coil, positioned within the landing gear axle. The sensor’s exterior features four equally spaced poles with machined teeth around its circumference. A soft-iron exciter ring, also toothed on its internal surface, is mounted within the wheel hubcap and rotates concentrically around the sensor. As the wheel turns, the relative motion between the sensor poles and exciter ring teeth causes periodic variations in the magnetic circuit’s reluctance. These variations modulate the magnetic flux passing through the pickup coil, inducing an alternating current whose frequency is directly proportional to wheel rotational speed. The associated control unit then converts this variable-frequency AC signal into a proportional direct-current (DC) voltage for integration into the braking control logic. A typical internal arrangement of a wheel-speed transducer is shown in Figure 3 [18,19].

**Figure 4:**
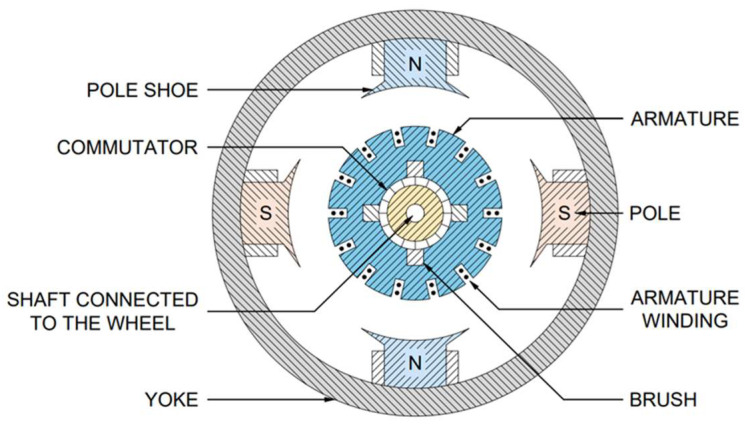
A schematic of a DC wheel-speed transducer.

In DC-based systems, the sensor functions as a small permanent-magnet DC generator, producing an output voltage proportional to the rotational speed of its armature. This arrangement eliminates the need for an AC-to-DC conversion stage within the control unit and reduces susceptibility to interference from stray induced voltages. The armature shaft is fitted with a blade driven by a bracket attached to the wheel hubcap, ensuring synchronous rotation with the wheel refer Figure 4. Output levels typically average approximately one volt per 16 km/h (10 mph) of wheel speed [19]. Overall, AC and DC wheel-speed transducers meet current antiskid needs, but improving low-speed accuracy and adding built-in health monitoring would strengthen next-generation aircraft system requirements.

##### Brake Temperature Probes

**Figure 5:**
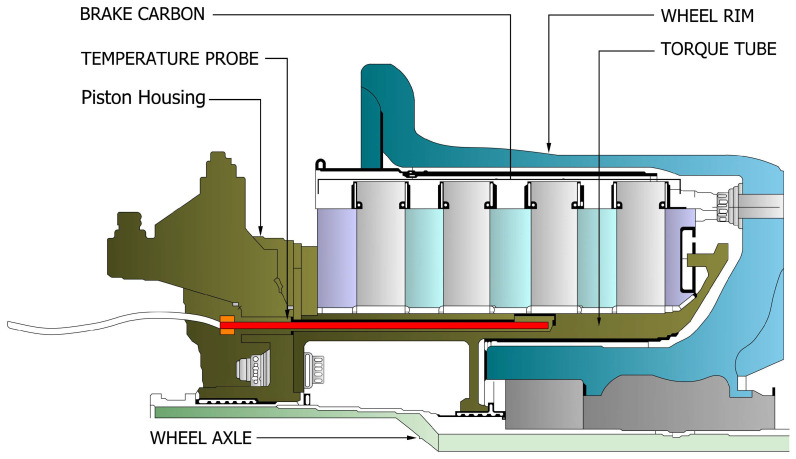
Brake temperature probe location. Diagram based on reference [21].

Brake Temperature Monitoring Systems typically use resistance temperature detectors to estimate brake stack temperature. Resistance temperature detectors are temperature sensors that work by measuring the change in a material’s electrical resistance, most often platinum, as the temperature changes [20]. Commercial aircraft brake temperature monitoring systems typically cover a broad measurement range from (−100 °C) to (+815 °C) [16]. Brake temperature strongly influences carbon-brake wear mechanisms. On most large civil aircraft, the brake-temperature probe is installed near the center of the heat pack as shown in Figure 5 to provide a representative brake stack temperature measurement that supports hot-brake advisories to the pilot, retraction of the landing gear, and dispatch decisions aimed at preventing tire or wheel overheating. Because the temperature probe tip is typically not in direct contact with the carbon disks (a small gap is present), the indicated temperature lags the true carbon disk temperature due to heat-soak effects and heat radiating across the gap [21]. Accordingly, landing gear health-monitoring functions that depend on brake temperature such as hot-brake detection, cooling-time estimation, and brake drag detection require measurement hardware and algorithms that account for this delay to ensure timely and accurate inputs.

As illustrated in Figure 5, the brake carbon forms the primary friction and heat generation element of the brake stack, while the temperature probe is positioned adjacent to the carbon discs through the piston housing in the torque tube to sense brake thermal state. The piston housing and torque tube provides structural support to the temperature probe. The wheel axle forms the central mechanical interface between the wheel and brake assembly and the landing gear, transmitting loads during ground operation. The surrounding wheel rim, shown in blue, encloses the brake assembly supporting the tire.

##### Tire Pressure Transducers

**Figure 6:**
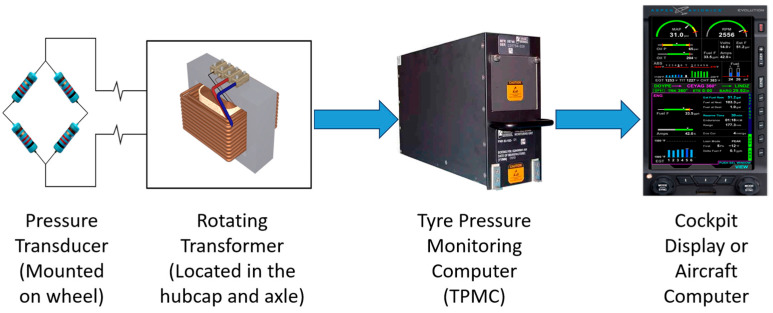
Tire pressure monitoring system (TPMS)—typical architecture.

An aircraft tire pressure monitoring system (TPMS) electronically measures and reports tire inflation pressure. Implementations range from systems that transmit data to the flight deck to solutions intended solely for ground-based maintenance use. Broadly, three architectures are described in the literature: (i) valve-stem gauge/inflation valve assemblies, where a direct reading gauge replaces the standard inflation valve, enabling maintenance crews to verify pressure during preflight walk-arounds [22]; (ii) handheld reader systems, where a passive wireless sensor embedded in the valve stem is interrogated by a portable reader for maintenance checks [23]; and (iii) cockpit-displayed systems, where a pressure sensor is mounted on the wheel directly senses tire pressure, while an electronics module at the wheel-hub provides transducer excitation and conditions the signal. The measured output is digitized and transmitted across the axle via a rotating Transformer to a processor (e.g., tire pressure indicating unit), which decodes the data, compares actual pressures with preset thresholds (both absolute and cross-axle differential), and commands cockpit alerts as required. Tire pressures can also be displayed to the flight crew on demand; refer Figure 6 for a typical TPMS architecture [22]. Building on these concepts, TPMS has become widely available as an integrated option, with a prevalent example being Crane’s SmartStem^®^, a passive wireless pressure/temperature sensor that replaces the inflation valve stem and communicates via an in-axle transceiver to a TBMU [23]. Reported benefits include increased tire life, reduced line-maintenance burden, and elimination of wiring on rotating hardware, making these systems a compelling alternative to fully wired implementations.

##### Brake Wear Indicators

Brake wear is the principal driver of brake-unit removal in airline operations. During daily line maintenance and pre-departure inspections, mechanics assess brake condition on each main-gear wheel. Most transport-category aircraft employ visual wear-indicator pins to gauge remaining carbon heat-stack thickness and thus brake serviceability. Inspection is performed with the brakes applied (to seat the stack), and the exposed length of the wear pin is compared against the manufacturer’s limits. For example, on single-aisle civil transport aircraft such as Airbus A320/Boeing 737 class aircraft, following installation of a new heat pack, the indicator typically measures about 49.6–50.4 mm with brakes applied; this dimension decreases as the stack wears. A brake is considered serviceable while the indicated length remains above the published minimum; when the indication reaches the limit, the unit is removed and replaced [12,24]. Because wear pins provide an immediate, tool-free indication, they are widely used for daily checks, with more detailed measurements reserved for scheduled maintenance.

Modern wide-body aircraft such as the Boeing 787 Dreamliner and Airbus A350 employ sensor-rich architectures that record condition and health parameters throughout service life, including within the brake system. In modern aircraft, electric brakes use electromechanical actuators to apply clamping force to the carbon friction stack, generating the shear work needed for deceleration; with routine operation, the stack is progressively worn, and its thickness diminishes. For rapid line checks, each brake incorporates two wear-indicator pins that provide a direct visual measure of remaining carbon. In parallel, the aircraft’s brake control electronics infer thickness by measuring actuator position (stroke) with the brakes applied and mapping this to calibrated geometry. The resulting wear state can be downlinked via Aircraft Communications Addressing and Reporting System (ACARS) as a percentage of the original stack thickness to support condition-based maintenance. Minimum allowable thickness margins are maintained to ensure stopping performance and to enable efficient refurbishment at removal [25]. However, because heat, actuator backlash, and sensor drift can bias stroke-based wear estimates, operators should routinely cross-check the brake wear condition with wear-pin readings to prevent both false alarms and missed wear.

#### 2.2.2. Research and Emerging Sensing Approaches

##### Sensor-Integrated Wear-Pin Technologies for Brake Wear Monitoring

Brake wear sensing traditionally relies on physical wear pins, which require visual inspection. The patent in [26] improves this by integrating an electromechanical proximity sensor (e.g., reed switch) into the brake assembly to detect when the wear pin passes a preset threshold, indicating brake stack wear. Sensor output can be routed to diagnostic systems or cockpit indicators, enabling on-condition maintenance without disassembly. Variants using photo-optical or sonic sensors are also described, offering non-contact detection options. Proximity-based wear-pin sensing can enable on-condition maintenance, but without rigorous calibration to true stack thickness and demonstrated robustness to heat, debris, vibration, and electromagnetic interference (EMI), fixed thresholds may yield false positives/negatives and fall short of certification reliability targets.

##### Fiber Bragg Grating-Based Structural Load and Health Monitoring

Fiber Bragg Grating (FBG) networks, surface-bonded or embedded within landing gear structural components, have been demonstrated as an advanced method for real-time load, strain, and hard-landing detection [27]. By measuring wavelength shifts in reflected light, FBGs enable precise monitoring of multiple parameters such as vertical touchdown loads, braking torque, and side-loads during taxi and landing events. These systems offer inherent immunity to EMI, the ability to multiplex numerous sensing points along a single optical fiber, and negligible added mass, making them especially attractive for aerospace applications. Research by Iadicicco et al. further demonstrates the integration of FBG sensors into composite drag struts of aircraft landing gear, where performance closely matched finite-element predictions across loading up to 70 kN, reinforcing the feasibility of remote, real-time load measurement as an early-warning approach [28]. Field trials under the ALGesMo (Advanced Landing Gear Sensing and Monitoring) project have validated their robustness in harsh operational environments, including resistance to vibration, temperature extremes, and contamination. They go beyond immediate operational feedback, such sensing capabilities support weight-and-balance estimation, fatigue life assessment, and condition-based maintenance strategies, aligning with future goals for integrated health and usage monitoring systems in commercial aviation [27]. However, while the application of FBG sensing in landing gear has shown strong potential, its extension to wheels and brakes poses additional challenges, including exposure to extreme thermal gradients, abrasive brake dust, and rapid transient loads during braking—conditions that demand further material robustness, calibration stability, and cost–benefit justification before widespread adoption in commercial fleets.

##### Energy Harvesting and Wireless Power Solutions for Wheel and Brake Sensors

In the context of wheels and brakes, one of the principal barriers to advanced sensing is the need to power sensors located near rotating assemblies, where conventional batteries and wiring are impractical due to maintenance burden, safety concerns, and environmental exposure. Recent studies have investigated energy harvesting—both vibration-based and thermal—as well as wireless power transfer solutions to overcome this limitation [29]. For instance, rim- or axle-mounted vibrational energy harvesters have been proposed to supply continuous power to tire pressure monitoring systems [30], while thermoelectric modules have been explored to convert brake heat into electrical energy for onboard sensor nodes [31]. Additionally, heat-storage-powered wireless strain sensing concepts have been demonstrated in related transport applications, capable of sustaining measurement and telemetry functions in the harsh temperature cycles and dynamic loads typical of aircraft braking events [32]. These approaches promise to enable greater sensorization of wheel and brake systems, supporting real-time health monitoring without the logistical drawbacks of wired connections or frequent battery replacement, thereby aligning with broader trends toward autonomous, condition-based maintenance in aviation. Nevertheless, the practical adoption of such energy harvesting solutions will depend on proving long-term reliability under the extreme thermal, mechanical, and contaminant exposure of aircraft wheel and brake environments as well as demonstrating a clear maintenance and cost advantage over existing battery-powered or wired alternatives.

#### 2.2.3. Certification and Standard Landscape for Wheel and Brake Sensors

Wheel and brake sensors—such as TPMS, TBMS, brake wear sensors, and associated BSCUs—are subject to a comprehensive certification framework addressing environmental qualification, system safety, and operational compliance. A comparative overview in Table 2 highlights the close alignment between FAA and EASA certification pathways for W&B sensor systems while also indicating subtle procedural and document-structure differences that may influence system qualification strategies in multinational certification programs.

While the tires and brakes themselves may be certified under applicable Technical Standard Orders (TSOs) in the United States or European Technical Standard Orders (ETSOs) in Europe, the airborne electronics used in W&B monitoring must be qualified to RTCA DO-160G [33] or its European equivalent EUROCAE ED-14G [34], which specify environmental and EMI test procedures for airborne equipment operating under extreme thermal, vibration, humidity, shock, and Radio Frequency exposure conditions [35]. Passing DO-160G/ED-14G environmental tests is necessary but not sufficient; wheel and brake sensing must also demonstrate traceable performance, calibration stability, and design-assurance compliance (e.g., ARP4754A/ARP4761, DO-178C/DO-254) within the aircraft safety case. Otherwise, hardware may survive the environment yet deliver unreliable data or inadequate fault coverage in service.

From a systems engineering and safety perspective, these electronic components are developed and assessed in accordance with SAE ARP4754A for civil aircraft and system development processes [36] and SAE ARP4761 for safety assessment methodologies [37], including Functional Hazard Assessments (FHAs) and Failure Mode and Effect Analysis (FMEA). Both the Federal Aviation Administration (FAA) and the European Union Aviation Safety Agency (EASA) recognize these standards as acceptable means of compliance. FAA references them in relevant Advisory Circulars for airborne system integration [38], while EASA outlines equivalent guidance in its Acceptable Means of Compliance (AMC) documents for Certification Specifications for Large Airplanes (CS-25), particularly AMC 25.1309, which governs system design, failure conditions, and safety objectives [39].

Tire-specific operational and maintenance requirements are defined separately in the U.S., under FAA AC 20-97B, which provides guidance on the installation, inflation, inspection, and removal of aircraft tires [40], and in Europe, under the EASA Part-26 and associated AMC and Guidance Material (GM), which address continued airworthiness and operational safety measures for landing gear and W&B assemblies [41]. While these documents do not directly regulate sensor electronics, they define the operational safety framework in which W&B sensors must function, shaping the design and qualification criteria for such systems. Collectively, RTCA DO-160G/ED-14G, SAE ARP4754A, SAE ARP4761, FAA AC 20-97B, ETSOs, and EASA CS-25 AMC/GM guidance form the certification and standards landscape for W&B sensor implementation. Compliance ensures that installed sensors meet environmental durability requirements, integrate safely within aircraft systems, and operate without adversely affecting braking performance or flight safety.

An investigation of brake material properties such as thermal conductivity, specific heat, and brake wear/resistance influencing brake temperature behavior quantitatively is beyond the scope of this review. Similarly, publicly available quantitative reliability metrics for wheel and brake sensors under operational conditions are limited and often proprietary. This review focuses on sensing, heat-transfer mechanisms, and predictive frameworks rather than material optimization.

## 3. Condition Monitoring Techniques in Aerospace

In the aerospace domain, condition monitoring denotes the continuous or periodic acquisition and analysis of system data to determine current condition (health), detect incipient faults, and support condition-based maintenance (CBM) decisions within the operator’s maintenance program. Formal definitions and process vocabularies are collated in ISO 13372/13379/13381, which separate data interpretation/diagnostics from prognostic estimation and emphasize traceable procedures, uncertainty management, and reporting conventions [42,43,44]. Within the broader airline maintenance context, CBM processes are evaluated against SAE JA1011 criteria for Reliability-Centered Maintenance, while SAE JA6268 provides design considerations for Prognostics and Health Management (PHM) systems deployed on aircraft [45,46].

Integrated Vehicle Health Management (IVHM) provides the primary capability for monitoring and managing aircraft system health, within which CM and its decision-focused extension, CBM, aimed at improving availability and reducing maintenance, repair, and overhaul costs. Authoritative frameworks from SAE and NASA describe IVHM as a life-cycle capability spanning onboard acquisition, real-time/near-real-time analytics, and ground-based decision support. Those underline benefits such as earlier fault detection, reduced no-fault-found events, and optimized maintenance scheduling [47,48]. In the research literature, IVHM is positioned as the organizing layer that fuses heterogeneous evidence (models, data, and reasoning) for operational decisions, with recent surveys detailing practical challenges and opportunities for aerospace applications [49].

Diagnostics addresses fault detection, isolation, and explanation (root-cause reasoning) using rule-based, model-based, or data-driven techniques [50], while prognostics estimates future degradation and remaining useful life (RUL) to support intervention before loss of function [51]. Reviews of machinery health monitoring and aerospace IVHM practices have formalized best-practice guidance in feature engineering, model selection, validation protocols, and performance metrics for both diagnostic and prognostic tasks [52,53]. Early aerospace implementations further demonstrate how prognostic layers added to legacy diagnostic architectures can materially improve CBM effectiveness [54]. Program-level exemplars, such as the PHM capabilities developed for the Joint Strike Fighter (JSF), highlight the system-engineering, logistics, and certification considerations required to transition these methods into operational service [55].

These concepts are directly relevant to landing gear wheels and brakes, where braking events produce high, transient thermal and mechanical loads that persist into multiple phases of landing gear operation. Effective CM for W&B therefore should be centered on (i) reliable sensing (embedded/remote) and data pathways, (ii) diagnostic logic that respects phase-dependent physics (e.g., heat soak-back into rim/tire), and (iii) prognostic models that translate observed usage/temperature history into actionable estimates of remaining margin and turnaround constraints. Section 2.2.1. provided an in-depth discussion of embedded sensors integrated within wheel and brake assemblies. The subsequent section extends this perspective by examining remote sensing methodologies employed across the aerospace industry.

### 3.1. Remote Sensing Methods

In condition monitoring, remote sensing methods encompass non-contact or non-intrusive techniques such as optical vision, acoustic/ultrasonic, radar, terahertz imaging, and infrared thermography that enable condition assessment without embedding sensors into structural or mechanical components. These methods align with established non-destructive testing practices and mitigate the invasive footprint associated with embedded systems [56].

In contrast, embedded CM systems such as the Aircraft Condition Monitoring System (ACMS) are typically integrated during manufacturing, and their retrofitting into legacy aircraft involves significant complications, including sensor installation, wiring, and certification burdens [57]. The airworthiness and certification frameworks in civil aviation are stringent and require complete specification and verification of system behavior before deployment, which further constrains the introduction of new embedded hardware or autonomous sensor systems post-certification [58,59].

Consequently, remote sensing emerges as a strategically advantageous alternative, offering greater deployment flexibility, particularly to avoid retrofitting legacy platforms and enabling expedited condition monitoring upgrades without modifying existing hardware or triggering extensive re-certification efforts on aircraft.

#### 3.1.1. Optical and Vision-Based Remote Sensing

Optical and vision-based sensing techniques are increasingly applied in aerospace condition monitoring as non-intrusive approaches to detect structural and system anomalies. Vision-based methods include digital image correlation, which has been widely reviewed for its ability to capture displacement and strain fields with high accuracy [60]. In addition, high-speed and multispectral imaging have been employed to detect cracks, corrosion, and deformation in metallic and composite structures during inspection cycles [61]. Laser-based approaches such as laser vibrometry and laser ultrasonics enable remote measurement of vibration signatures, displacement, and subsurface defect detection without physical contact [62,63]. Complementing these methods, fiber-optic sensors have been investigated for aerospace applications owing to their low weight, immunity to electromagnetic interference, and capability for distributed strain and temperature measurements [64]. Collectively, these optical and vision-based approaches contribute significantly to structural health monitoring by reducing reliance on intrusive embedded hardware and offering scalable integration for both metallic and composite airframes.

#### 3.1.2. Acoustic/Ultrasonic Remote Monitoring

Wave-based sensing techniques in aerospace condition monitoring broadly include Acoustic Emission (AE) and Ultrasonic Testing (UT), which, while often grouped together, operate on different principles. AE is a passive technique that captures stress waves generated by damage events such as crack initiation, fiber breakage, delamination, or fatigue, enabling real-time detection in otherwise inaccessible regions [65]. It is among the most mature non-contact CM approaches in aerospace, with demonstrated effectiveness in the aerospace industry [66]. Subsequent applications have extended to helicopter drivetrains through AE-based health and usage monitoring systems (AE-HUMS), highlighting its operational value [67]. While AE provides valuable early-warning capability, its reliability depends heavily on advanced signal processing and noise discrimination, especially within the complex acoustic environments of aircraft [65].

By contrast, UT is an active technique in which externally generated ultrasonic waves introduced via piezoelectric or laser sources are propagated through materials, and reflections, scattering, or attenuation are analyzed to reveal structural integrity. UT has been widely applied to thickness gauging, delamination detection in composites, and adhesive bond inspections in aerospace [68]. Recent developments in laser ultrasonics further extend UT to non-contact applications, enabling the detection of subsurface defects such as cracks and delamination without physical coupling [63]. Collectively, AE offers sensitivity to damage initiation, whereas UT provides controlled defect characterization, making the two approaches complementary within integrated CM frameworks for aircraft.

#### 3.1.3. Electromagnetic Based Sensing

##### Radar and Microwave Sensing

Radar and microwave sensing have been adopted for monitoring both environmental hazards and structural conditions in aerospace [69]. Ground-based and polarimetric radar systems can remotely detect in-flight icing by identifying hydrometeors in clouds, offering critical situational awareness for flight safety [70]. The Ground-based Remote Icing Detection System (GRIDS) developed by the National Center for Environmental Prediction (NOAA) demonstrates how radar and radiometer synergy support aircraft icing forecasts near airports [71]. While their primary application lies in environmental monitoring, the challenge remains in translating such data streams into actionable aircraft health intelligence, highlighting the need for tighter integration between atmospheric sensing and onboard condition-based maintenance frameworks.

##### Radiation-Based Techniques

Radiation-based techniques encompass a wide range of non-destructive evaluation (NDE) methods, including X-Ray, gamma, and neutron imaging, each providing subsurface diagnostic capability for aerospace structures [56]. Within this spectrum, terahertz (THz) imaging has recently emerged as a promising complementary tool. Unlike conventional optical methods limited to surface inspection, THz imaging operates in the 0.1–10 THz band and enables identification of delamination, inclusions, and moisture ingress within layered composites [72,73]. Advances in terahertz time-domain spectroscopy further demonstrate its potential for the real-time detection of multi-delamination defects in glass-fiber-reinforced composites [73]. As modern aircraft increasingly adopts composite airframes, THz imaging is gaining traction as a non-intrusive inspection method that complements established radiation-based approaches [74]. However, despite these advances, the widespread adoption of radiation-based and THz techniques in operational aerospace remains constrained by factors such as equipment cost, penetration depth limitations in conductive materials, and stringent safety requirements, underscoring the need for further maturation before integration into routine aircraft health-monitoring workflows.

#### 3.1.4. Infrared Sensing (Thermography)

Infrared (IR) thermography is a passive, non-contact technique that visualizes surface temperature fields through detection of naturally emitted thermal radiation. Its ability to provide rapid, wide-area temperature mapping makes it particularly relevant to aerospace condition monitoring, where thermal behavior is closely linked to structural integrity. IR methods have been applied to composite airframes for detecting delaminations, disbonds, and moisture ingress [75]. Recent work has extended these capabilities to landing gear systems, where IR thermography has been used to observe wheel and brake temperature decay immediately after landing [76,77]. Further studies highlight how spatially resolved thermal imagery can reveal component-level gradients across the heat pack and wheel structure, supporting empirical estimation of cooling rates and post-landing thermal behavior [76,77].

A key challenge in quantitative thermography is emissivity (ε), which varies significantly across aerospace materials and surface finishes, such as aluminum alloys, carbon composites, titanium, and tire rubber leading to potential under or overestimation of true temperatures if uncorrected [78]. To mitigate these effects, practitioners rely on emissivity calibration, reference targets, or published emissivity tables [79,80], though low-emissivity metals remain sensitive to reflected environmental radiation.

Despite these constraints, thermography remains one of the most practical techniques for non-intrusive thermal assessment in aerospace. Its advantages include portability, rapid data acquisition, and suitability for both in-service and maintenance-bay inspections. However, limitations such as emissivity dependence, environmental sensitivity, and surface-only penetration must be considered when interpreting results [75]. Overall, IR thermography provides a valuable capability for monitoring thermal behavior in safety-critical systems, especially for wheels and brakes, where spatial temperature fields underpin both operational safety and predictive maintenance strategies.

**Figure 7:**
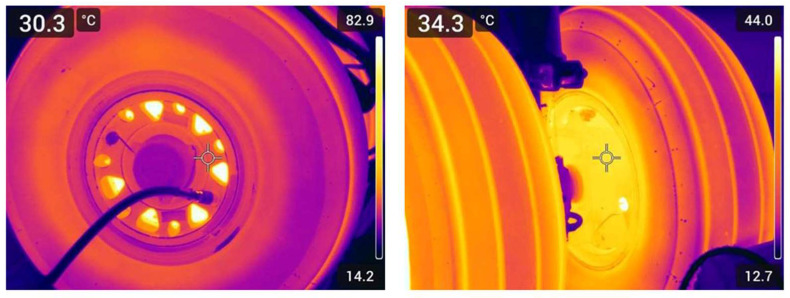
Representative infrared thermograms of Cranfield University Saab 340B aircraft W&B assembly post-landing.

To ground the above discussion, Figure 7 presents representative infrared thermograms of the main landing gear wheel and brake assembly of the Cranfield University Saab 340B aircraft acquired shortly after landing. The thermograms illustrate the pronounced spatial non-uniformity of surface temperatures across the brake, wheel web, rim, and adjacent tire regions, which cannot be inferred from conventional embedded sensors alone. Distinct temperature gradients are visible across geometrically and thermally coupled subcomponents, highlighting the complex post-landing thermal state of the wheel–brake assembly. While such thermograms provide valuable spatial insight into surface-level thermal behavior, they remain limited to surface observation and do not independently resolve internal conductive pathways or decouple conduction–convection interactions.

### 3.2. Intelligent Prediction Models

#### 3.2.1. Fundamentals: Artificial Intelligence (AI), Machine Learning (ML), and Neural Networks (NNs)

AI refers to computational methods designed to perform tasks that would normally require human reasoning or decision-making. Within AI, ML represents a key subfield that develops models capable of learning patterns or relationships directly from data. The principal paradigms of ML include supervised learning, such as regression or classification; unsupervised learning, such as clustering or anomaly detection; semi-supervised and self-supervised approaches; reinforcement learning; and more recent extensions such as transfer, online, and federated learning. NNs form another important component of AI, providing a means of approximating complex nonlinear functions through layered structures of interconnected processing units. When these networks are extended to include many layers and large numbers of parameters, the approach is termed Deep Learning (DL). Well-known examples include Convolutional Neural Networks (CNNs) for image analysis, Recurrent Neural Networks and Long Short-Term Memory (LSTM) networks for time-dependent data, Gated Recurrent Units as a streamlined alternative to LSTMs, and Temporal Convolutional Networks and Transformer architectures for sequence modeling [81,82]. Although AI encompasses a wide spectrum of techniques, its practical impact on engineering prediction tasks depends less on the taxonomy of methods and more on the ability to align ML, NNs, and DL architectures with the specific structure, scale, and constraints of the problem domain.

In the context of engineering prognostics, ML methods, including neural network-based approaches such as DL, are typically applied in three principal ways. First, they are employed to learn mappings between multi-modal sensor data, operational parameters, and contextual variables in order to predict target outputs such as component temperatures. Second, they provide the capability to detect anomalies and forecast the progression of abnormal conditions, thereby supporting early fault identification. Third, they are developed to estimate remaining useful life or remaining operational margins, offering quantitative insights that directly inform maintenance planning and condition-based maintenance strategies. Collectively, these applications position ML, NNs, and DL as essential enablers of predictive modeling in safety-critical domains such as aerospace [50,51]. However, the practical value of ML, NNs, and DL in aerospace prognostics ultimately depends not only on their predictive accuracy but also on their ability to deliver interpretable, certifiable, and operationally robust insights within the constraints of safety-critical environments.

#### 3.2.2. Approaches for Developing Prediction Models Using ML

Prediction models in engineering aim to convert historical records, sensor measurements, and operational context into reliable estimates of future states and their associated uncertainties. Within this process, several classes of ML techniques can be distinguished, each offering strengths depending on the nature of the input data and the structure of the prediction task.

##### Tabular Learners (Feature-Based Learners)

Tabular learners, including random forests, gradient-boosting machines, and Support Vector Machines, form a widely used class of models for structured, engineered features derived from physical understanding or operational context (e.g., aircraft mass, wind conditions, runway characteristics, phase of flight). Such models have long been recognized as effective baselines in aerospace diagnostics and prognostics, where their behavior can be readily interrogated and validated [51]. Recent work in brake-temperature prediction further demonstrates the suitability of these learners when combined with carefully engineered physical variables and operational signals, showing that feature-based models can provide stable, interpretable performance in safety-critical thermal-monitoring applications [83].

##### Sequence Models

For time-dependent processes, sequence models offer enhanced capability. Architectures such as LSTM networks, Temporal Convolutional Networks, and Transformer-based models are designed to capture dependencies across time, making them particularly useful for multi-horizon forecasting of temperatures, loads, or failure risks [51,84]. These models can represent the hysteresis and lagging effects that are common in thermal and mechanical systems.

##### Image-Based Models

When spatially rich data are available, such as infrared thermograms or thermal image sequences, image-based models become relevant. CNNs, including their three-dimensional variants (3D-CNNs), can learn localized and global thermal patterns, detect anomalies, and infer how these patterns evolve under operational stresses [85,86,87]. This capability allows for the extraction of features that may be difficult to define explicitly through hand-crafted engineering rules.

##### Cross-Cutting Considerations (Good Practices in Model Training)

In all cases, deploying prediction models in aerospace requires rigorous attention to uncertainty quantification and model validation. Differentiating between aleatoric (data-driven) and epistemic (model-driven) sources of uncertainty ensures that predictions remain both accurate and reliable under variable operational conditions. Calibration techniques such as conformal prediction are increasingly recommended to produce well-bounded confidence intervals suitable for safety-critical decision-making. Furthermore, evaluation protocols must avoid optimistic bias by adopting temporally aware validation strategies, including blocked or rolling-window time splits rather than random partitions, as emphasized in leading CM/PHM reviews [50,51,52].

Machine Learning prediction models can be grouped according to the type of data they are designed to process. Table 3 summarizes the principal approaches discussed, linking each method to its typical data input and highlighting the key advantages for aerospace prognostics.

Ultimately, the effectiveness of ML prediction models in aerospace does not hinge solely on selecting a particular algorithm but rather on aligning the modeling approach with the structure of the available data and enforcing rigorous validation and uncertainty quantification to ensure operational reliability in safety-critical contexts.

#### 3.2.3. Physics-Informed and Hybrid Learning

Traditional data-driven ML models are often limited by their dependence on large, high-quality datasets, and they may struggle to generalize under unseen operating conditions. To address these challenges, physics-informed and hybrid learning approaches have been proposed, combining the adaptability of ML with the robustness of physical principles. In particular, Physics-Informed Neural Networks incorporate governing equations such as the heat conduction equation, together with initial and boundary conditions, directly into the model’s training process. By embedding these constraints into the loss function, Physics-Informed Neural Networks ensure that the learned thermal fields remain physically consistent, even when trained on sparse or noisy data [88,89,90]. In doing so, they combine the efficiency of data-driven models with the extrapolation power of physical knowledge.

These hybrid strategies are particularly valuable when extending predictions across different aircraft variants, mission profiles, or environmental conditions, where purely data-driven models may otherwise be overfit to limited training distributions. Moreover, their computational efficiency makes them suitable for integration into IVHM frameworks and ACMS, where runtime constraints and certification requirements demand models that are both reliable and interpretable.

#### 3.2.4. Validation, Uncertainty, and Deployment

The reliability and operational acceptance of prediction models in aerospace depend not only on their accuracy but also on the rigor of their validation strategies and the credibility of their uncertainty estimates. CM-oriented reviews stress that evaluation must extend beyond simple random train–test splits, recommending instead validation protocols that capture variations across held-out fleets, airports, and operational environments to ensure genuine generalization rather than overfitting to a single dataset [50]. PHM research further emphasizes the need for temporally structured validation, such as blocked or rolling-window schemes, to prevent information leakage and to provide a realistic assessments of predictive performance under real operational timelines [52]. In addition, phase-aware scoring approaches, where model performance is evaluated separately for landing, taxi, and turnaround, are essential for systems whose behavior is strongly influenced by the operational phase. This requirement is particularly relevant for wheels and brakes, where thermal evolution and cooling dynamics depend closely on flight-segment transitions and environmental context [91,92].

A second requirement concerns uncertainty calibration, which ensures that predictive distributions correspond to meaningful confidence levels in real operational settings. This is especially critical in decisions such as brake cool-down release or assessing thermal margins, where under- or overestimation of uncertainty can directly affect safety. PHM research stresses that uncalibrated models may achieve low point-prediction error while still providing misleading confidence bounds, thereby reducing operational trustworthiness [50]. To address this, methods such as conformal prediction, Bayesian calibration, and ensemble-based uncertainty estimation are increasingly recommended for safety-critical applications, as they provide well-bounded intervals and maintain validity under distributional shifts [52,91,92].

Operational deployment within IVHM or ACMS frameworks introduces additional requirements relating to data governance, model-drift management, and certification. Certification guidance for adaptive or learning-enabled systems highlights the need for algorithmic transparency, update traceability, and verifiable artefacts demonstrating robustness across diverse operational conditions [58]. Broader aerospace assurance frameworks similarly emphasize the importance of documenting model assumptions, data lineage, and verification evidence to support engineering acceptance [59]. To be operationally trusted, models must also produce interpretable outputs such as feature attributions, sensitivity analyses, or key-driver identification that allow maintenance engineers to confirm that model reasoning aligns with physical understanding. When such calibrated and interpretable outputs are used to inform maintenance-release decisions, including brake cool-down times, they help ensure that predictive models can meet the rigor expected in regulated aerospace environments.

From an integrated condition-monitoring perspective, the performance of intelligent prediction models is shaped by how effectively sensing, physics, and data-driven learning are brought together. The sensing methods discussed in Section 2.2.1 provide operational measurements of key wheel and brake state variables, such as brake temperature, wheel speed, and tire pressure, offering localized, practically accessible indicators of thermal loading during operation. The heat-transfer mechanisms described in Section 4, including conduction within the brake stack and wheel, convection during roll-out and taxi, and radiation following gear retraction, establish the physical constraints that affect temperature evolution across different operational phases. When AI and ML models are trained using such sensor data and are augmented by physics-informed features or constrained by heat-transfer principles, they can learn relationships that support inference beyond directly instrumented locations. This capability is particularly important for condition monitoring and non-destructive evaluation, where dense sensor deployment is often impractical and spatial–thermal states must be inferred from limited measurements. The gaps identified in Section 5 further suggest that progress in this area will depend not on advances in sensing, modeling, or AI/ML in isolation but on their coordinated integration to address data sparsity, improve generalization under unseen operating conditions, and enable operationally deployable health-monitoring frameworks. Taken together, the literature indicates that the primary bottleneck is not predictive algorithms or thermal modeling capability, but the limited availability of spatially resolved, real-flight thermal data needed to link sensing, physics, and AI/ML based prediction.

## 4. Heat Transfer Dynamics in Aircraft Wheels and Brakes

### 4.1. Scope and Relevance

During landing, and particularly during a rejected take-off, the landing gear wheels and multi-disc carbon brakes convert a significant fraction of the aircraft’s kinetic energy into heat within the brake stack. This results in rapid and highly non-uniform temperature rises at the friction interfaces which subsequently propagate through the discs, pressure plates, torque path, and wheel components, producing a transient thermal field that persists well beyond the braking event [93]. These temperature transients directly shape both design margins determining the required brake size and the thermal protection needed for the wheel, tire, and fuse plugs and the operational limitations imposed on airlines. Regulatory and certification guidance defines the maximum brake energy (MBE) that the system may safely absorb and specifies the maximum quick-turn weight (MQTW) permissible before the next departure [94,95]. Complementary modeling and operational studies show how these limits translate into required cool-down times, reflecting the relationship between post-braking thermal decay and dispatch readiness [96,97,98]. The core challenge lies in reconciling these design and turnaround constraints with the inherently space–time-varying thermal field methods that rely on averaged temperatures or single-sensor readings risk missing local peaks in the brake stack or rim, thereby undermining the protective margins assumed by MBE and MQTW criteria.

The temperature rise within the wheels and brakes assembly is governed by three coupled elements: (i) frictional generation at the disc–pad interface, including flash temperatures, contact non-uniformity, and associated transient conduction within the friction ring [99,100]; (ii) a time-varying heat–partition ratio between rotor and stator that depends on contact pressure, sliding speed, surface condition, and the thermal properties of carbon/C–SiC (carbon-fiber-reinforced silicon carbide composites) (often estimated from effusivity ratios and refined via inverse identification) [101,102,103]; and (iii) post-event cooling by conduction through the torque path into the wheel/tire, forced convection to the airstream and through internal ventilation passages during roll-out/taxi, and thermal radiation in the enclosed wheel-well environment when retracted [99,102,104]. Because each mechanism varies strongly with operating state—vehicle speed, brake pressure, surface condition, and material properties—and published values rarely include quantified uncertainty, relying on empirical estimates is unreliable for analysis and decision-making. A defensible LR-informed workflow is to (a) impose aircraft-specific boundary conditions for each operational phase, (b) identify uncertain parameters (e.g., partition factor, time-varying local convective heat-transfer coefficient) using inverse methods with bench/flight data (brake temperature monitoring systems, IR thermography), and (c) validate on-aircraft while propagating uncertainty into MBE/MQTW cool-down predictions and maintenance decisions for wheels and brakes.

#### 4.1.1. Conduction Paths

**Figure 8:**
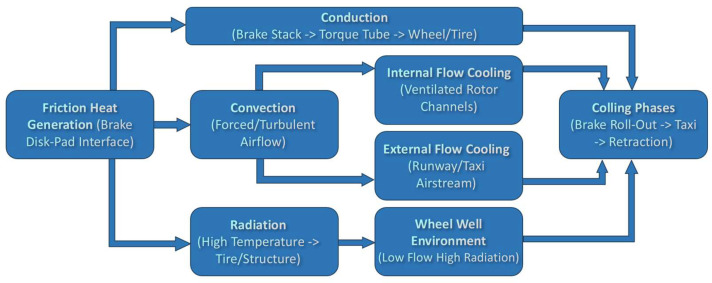
Aircraft wheel and brake heat transfer pathways.

The dominant W&B heat transfer pathways and modeling methods are summarized in Figure 8. Immediately after a brake event, frictional heat built up in the carbon stack conducts through the pressure plate, torque tube, wheel hub, and rim (the “torque path”). Because carbon–carbon (C/C) heat packs are anisotropic (higher in-plane than through-thickness conductivity), hot spots at the friction ring can migrate axially and then radially into metallic hardware on time scales of minutes, even as runway convection falls away [103,105]. Coupled brake–wheel models and sensor-hardware analyses show this soak-back clearly: the brake cools while the wheel rim and tire continue to warm, so wheel/tire peaks may occur long after the peak in internal brake components [94,106]. This is operationally relevant because 14 CFR §25.735 (airworthiness standards for brakes and braking systems on transport-category airplanes) requires an “over-temperature burst prevention” means, typically fusible plugs, and FAA/EASA guidance expects high-energy demonstrations to show that wheel/tire and nearby structure remain protected under all aircraft operational envelop [95]; radiative paths would otherwise raise rim temperatures, and heat shields (chin rings) are installed to increase thermal resistance and reduce direct view of hot surfaces, demonstrably lowering rim/tire heating in service [107].

Conduction analyses must incorporate (i) anisotropic carbon properties, (ii) contact conductance at interfaces (backplate, torque-tube splines), and (iii) the wheel/tire thermal mass and boundary conditions (inflation gas, rim thickness) [94,106]. Validated finite-element studies of aircraft wheel–brake assemblies show that neglecting these couplings can under-predict rim temperatures and fuse-plug margins.

#### 4.1.2. Convection—Internal and External

During the landing roll-out and taxi phases, convection becomes the dominant mechanism governing heat removal from the wheel–brake assembly. External convection arises from the forced cross-flow generated by the runway and taxi airstream, which sweeps over the wheel rim, and exposed brake surfaces. At the same time, internal convection is driven by the pumping action of the rotating brake rotor, which induces through-flow within the ventilated passages and inter-disc gaps. This circulation draws cooler air into the brake cavity and expels heated air radially outward, forming the internal pathways illustrated in Figure 8.

The behavior of these flow fields is well characterized in the broader rotating-disk and rotor–stator cavity literature. This body of work establishes how Reynolds number, flow regime, and geometric configuration influence local convective heat-transfer coefficients and corresponding Nusselt-number distributions, and these correlations are routinely applied in aircraft brake thermal models [108]. In particular, these studies show that convective heat transfer generally increases with radial position due to rising tangential speed and that cavity through-flow enhances cooling effectiveness at the outer annulus.

Computational Fluid Dynamics (CFD) investigations further demonstrate how features such as vane count, vane angle, and inter-disc gap ratio influence internal flow development. Narrow, well-defined gaps tend to promote higher levels of shear and turbulence, improving heat transfer, whereas overly large gaps weaken the rotor-pumping effect and reduce local cooling. CFD results also highlight that certain cavity geometries can generate recirculation zones near the hub or behind misaligned vanes, leading to reductions in the local Nusselt number and creating thermal non-uniformity across the brake rotor [109].

These spatial and temporal variations in the convective heat-transfer coefficient, h(r,θ,t), strongly influence the transient thermal response of the brake stack during roll-out and taxi. Consequently, phase-dependent convection models derived either from reduced-order correlations or from CFD solutions are used to supply boundary conditions for finite-element simulations. This approach supports assessments of brake cooling behavior, quick-turn readiness, and compliance with maximum brake energy and minimum-cooling-time requirements [100,109]. Through this integration of classical rotating-disk theory and aircraft-specific CFD studies, internal and external convection remain central to predicting post-landing brake temperatures.

Because convective capacity collapses strongly with speed proportional to velocity squared, taxi-phase cooling is far less effective than on-runway cooling, increasing the role of conduction/soak-back. Concept studies demonstrate that ducted wheel-well ventilation or modest forced flow can significantly increase brake cooling during ground operations with limited drag penalties [104]. For aircraft-specific predictions, boundary conditions should switch with phase (high-Reynolds-number cross-flow on roll-out → low-Reynolds-number external convection on taxi) and include internally pumped flows in ventilated rotors. Using generalized correlations without phase-aware updates tends to mis-estimate cooling times and quick-turn limits.

#### 4.1.3. Radiation and Wheel-Well Enclosure Effects

After gear retraction, bulk airflow inside the wheel-well bay is weak; the brake–wheel assembly therefore transitions from a convection-dominated setting to an enclosure-radiation problem, with conduction becoming comparatively less significant. In this configuration, the net heat exchange is set primarily by geometric view factors among the hottest brake surfaces (friction rings, back-plates), the wheel rim/sidewall, any heat shields/liners, and the bay structure [98]. As convective coefficients collapse with speed, radiative heat flux q″ scaling with σε(T^4^_hot_ − T^4^_sur_) (where σ—the Stefan–Boltzmann constant, ε—the surface’s total hemispherical emissivity, T_hot_—absolute temperature of the emitting surface, and T_sur_—absolute temperature of the surroundings) can govern the early post-retraction cool-down, especially for high-emissivity carbon surfaces; this is precisely the condition for which gray/diffuse enclosure analysis (radiosity–irradiation with accurate view factors) is recommended [110,111]. Transport-airplane guidance reflects the same hazard: for retractable-gear installations, fusible plugs are not a complete safeguard, and temperature indication should be provided where overheating could damage wheel-well equipment, i.e., the bay must be treated as a low-flow enclosure in the thermal analysis and monitored accordingly [98]. Mitigations target the enclosure physics. Heat shields/liners (chin rings) with low effective emissivity facing the brake reduce the radiative view to the rim/tire and cut the net exchange toward the wheel. This approach is documented in dedicated shield designs and service practices [107]. Where integration allows, wheel-well ventilation (small intake/extraction paths or ducted flow) is used to re-establish a modest forced-convection sink after retraction and accelerate cool-down of the enclosed assembly [104].

Post-retraction thermal models should include, at minimum, a gray/diffuse enclosure-radiation treatment with measured or effective emissivities for carbon friction elements and metallic (or shielded) wheel surfaces, plus a view-factor network that accounts for multiple reflections between closely spaced rings and shields [110,111]. Neglecting the enclosure term can under-predict rim/tire temperatures and understate wheel-well equipment exposure, in conflict with the over-temperature protection and indication expectations in the landing gear/retraction guidance [98]. For phases prior to retraction, the aircraft brake models should switch back to cross-flow and cavity convection boundary conditions. Open reviews and CFD/experiment studies on rotor–stator cavities provide phase-appropriate convection data that complement the enclosure-radiation treatment [108]. This highlights a methodological gap in current brake thermal models, while many prioritize convective heat transfer during ground roll, insufficient attention to enclosure-radiation physics post-retraction risks, underestimating component exposure, and overlooking design optimizations in shielding and ventilation strategies.

#### 4.1.4. Operational Phases and Cooling Timelines

Published research consistently shows that the relative importance of heat transfer mechanisms in aircraft wheels and brakes changes across operational phases. During the landing roll, high external flow velocities promote strong forced convection, both over exposed brake faces and through ventilated passages. Findings from rotating-disc cavity literature confirm Reynolds number scaling of convective coefficients [108], while recent full-scale simulations of brake discs for medium-sized passenger aircraft validate the dominant role of cross-flow convection immediately after braking [112].

As the aircraft slows into taxi, cooling effectiveness diminishes. Convective coefficients fall approximately with airspeed following Un (with n ∼ 0.5–0.8 depending on geometry) (where U air speed and n—exponent from standard Nusselt correlations), consistent with classical correlations [110]. Empirical investigations highlight that as convection weakens, conductive soak-back into the wheel rim becomes more influential, often producing delayed rim and tire temperature peaks well after the brake stack itself has begun to cool [94,106]. Airbus service documentation and flight-test evidence confirm that brake-temperature indications, frequently sourced from torque-tube or stator probes, may lag or under-represent the critical exposure of the rim and tire [7,113].

Once the gear is retracted, the cooling environment alters significantly. The wheel well becomes a low-flow enclosure in which radiative heat exchange dominates until surface temperatures drop below a few hundred degrees Celsius. Foundational radiation studies emphasize the necessity of accurate emissivity data and enclosure view-factor analysis [110,111]. Regulatory sources highlight this concern, noting that fusible plugs are not considered sufficient safeguards and that dedicated temperature indication and protection must be provided in the wheel well to ensure structural safety [95,97,98].

From an operational perspective, mitigation strategies are well documented. Manufacturers recommend phase-aware procedures, including the use of brake fans on regional and business aircraft, extended taxi cooling in hot-and-high conditions, or delayed gear retraction when thermal margins are limited [7,8,104]. Field trials and experimental studies further demonstrate that ducted wheel-well ventilation can re-establish modest forced-flow sinks and shorten cooldown times [104,112]. Collectively, the literature underline that brake thermal behavior is inherently phase-dependent, with convection, conduction, and radiation each prevailing at different points in the landing–turnaround cycle; however, moving from these empirical findings to reliable turnaround and certification tools requires dedicated modeling approaches, which are examined in the following section.

### 4.2. Thermal Modeling—Numerical, Empirical, and Hybrid Models

Thermal modeling of aircraft wheels and brakes has evolved as a structured means of translating empirical heat-transfer observations into predictive turnaround and certification tools. As highlighted in Section 4.1, conduction, convection, and radiation dominate at different phases of operation, necessitating modeling approaches that can capture these mechanisms in a unified way. Early studies relied on simplified empirical and semi-analytical approximations, typically assuming uniform surface heat fluxes and constant partition ratios. These offered rapid estimates suitable for preliminary decision-making but proved inadequate in resolving non-uniform interface heating, anisotropic conductivity of carbon–carbon composites, and delayed soak-back into the wheel rim [114].

With the growth of computational resources, numerical methods, notably FEM and conjugate CFD simulations, have become the dominant strand of research. FEM studies have demonstrated their capability to resolve transient temperature distributions in brake discs under varying braking pressures, accounting for thermoelastic coupling and hot-spot instabilities [115,116]. Conjugate CFD approaches have further advanced this field, capturing phase-dependent convection in rotor–stator cavities and around ventilated rotors, which strongly governs brake cooldown rates [108,117]. For aircraft brakes specifically, full-scale test bench investigations have been integrated with FEM/CFD models to identify frictional heat fluxes and validate thermal histories, confirming the critical role of soak-back and rim heating in certification assessments [113,118]. Radiation modeling has also been incorporated into aircraft-oriented simulations, where enclosure effects in the wheel well substantially influence post-retraction cooling [112].

Despite the computational fidelity of numerical methods, they are resource intensive and depend on accurate boundary conditions, often difficult to specify in airline operations. As a result, empirical methods continue to serve operational needs where turnaround cooling predictions must be made quickly. However, these often neglect second-order effects, such as rim heating and enclosure radiation [119]. To bridge this gap, hybrid frameworks have emerged, embedding CFD-derived heat transfer coefficients or inverse-identified fluxes into reduced-order models. Such strategies have shown promise in retaining numerical accuracy while reducing computational load [120,121]. This trajectory of research highlights a conflict between accuracy and practicality; while numerical models offer unparalleled fidelity in capturing coupled thermal phenomena, their limited operational applicability underscores the pressing need for validated hybrid approaches that can realistically integrate into airline maintenance and certification workflows.

#### 4.2.1. Numerical Models (FEM/CFD & Conjugate Multi-Physics)

The literature reveals a layered progression of numerical techniques developed to represent the coupled thermal phenomena in aircraft wheels and brakes. Three main categories are distinguished: (1) FEM-based transient conduction and thermo-elastic modeling, (2) CFD-informed conjugate aero-thermal modeling, and (3) radiation-integrated and inverse calibration methods.

##### FEM: Transient Heat Transfer and Thermo-Mechanical Response

Initial FEM efforts focused on axisymmetric or limited 3D conduction modeling, laying groundwork for understanding disc temperature rise under uniform heat fluxes [66]. These were expanded by more complex finite-element simulations that incorporated anisotropic conductivity, contact conductance dependent on brake pressure, and realistic torque-path conduction capturing the delayed temperature advance into the rim and tire [99,122].

Thermo-mechanical coupling via FEM allowed for simulation of localized hot-spot initiation and stress evolution, aiding assessment of brake durability and structural limits under repetitive braking cycles [116]. Studies of disc materials, including C/C composites, further revealed how material anisotropy and interface behavior affect temperature distribution and cooling rates [103].

Full-wheel models extending beyond brake discs have also been developed, such as lumped-parameter thermal models covering brake, axle, rim, and tire temperatures, and validated against experimental rig data illustrating trade-offs, for instance, when using carbon fiber re-enforced plastic rims [123]. FEM can show where hot spots form and how heat soaks into the rim and tire, but unless contact laws, anisotropic properties, and boundary conditions are measured and validated phase-by-phase against rig data, the model may fit one braking case yet give non-transferable predictions for other duty cycles or wheel/rim configurations.

##### Conjugate CFD and Aero-Thermal Integration

Convection modeling has progressed from reliance on rotating-disk correlations to conjugate heat-transfer (CHT) CFD that resolves external cross-flow and internal ventilation through vaned passages and rotor–stator cavities. Modern studies explicitly compute impingement footprints, vane-channel pumping, recirculation pockets, and the resulting spatially varying heat-transfer coefficients h(r,θ) on the rotor/stator faces [124,125]. When these CFD-derived boundary conditions are coupled to solid conduction in the carbon stack and torque path (FEM/FEA), the combined models deliver time-resolved brake cooling predictions that reflect phase changes from high-speed roll-out to taxi (falling Reynolds number and internal through-flow) [126,127]. Open CFD/experiment work on ventilated rotors confirms how vane count/angle, inter-disc gap, and through-flow rate reshape local h and thus surface temperature decay. Higher through-flow increases the mass of air driven through the rotor passages, raising local shear and turbulence levels and thereby enhancing convective heat transfer at the disc surfaces. Conversely, insufficient through-flow weakens the pumping action, promotes recirculation in low-momentum regions, and reduces the local heat-transfer coefficient, particularly near the hub and behind vane channels, findings that have been used to calibrate reduced-order cooling models for operations [128].

Recent co-simulation/CHT workflows (tight or staggered coupling between CFD and FEM) show that resolving the fluid–solid interface in time improves both the magnitude and timing of predicted temperature peaks on the friction rings and backplates while remaining computationally tractable for design iteration [126,127]. Optimization-oriented CFD studies demonstrate that comparatively small changes in vane geometry and gap ratio (s/R) can yield material improvements in cooling effectiveness and hot-spot suppression [127,128]. CHT CFD can capture detailed convection and improve peak-timing forecasts, but unless wall models, flow boundary conditions, and phase-specific validation are accurate, conclusions about vane geometry or gap ratio and any reduced-order models (ROMs) built on them may be overconfident and may not generalize.

##### Radiation Coupling and Inverse-Model Calibration

After gear retraction, weak bay airflow elevates the importance of enclosure radiation in the wheel well. Conjugate solvers that embed gray/diffuse radiosity with accurate view-factor networks (including multiple reflections) better reproduce rim and bay-structure temperatures than simplified emissivity-only models, particularly during the early post-retraction interval when carbon surfaces remain hot [129,130].

To align models with measured response, inverse heat-conduction techniques have been used to estimate unknown heat flux histories at the friction interface and to identify effective parameters (partition factor, contact conductance, phase-dependent h(t)) from full-scale or flight-like benches. Notably, nonlinear inverse identification from embedded thermocouples/IR data recovers space and time-dependent heat fluxes, which then drive improved predictive fidelity in aircraft-scale disc assemblies [131,132]. If credible post-retraction and turnaround forecasts truly rest on a multi-physics model—frictional generation → anisotropic C/C conduction → phase-aware convection → enclosure radiation—calibrated via inverse methods, then a tightly coupled CHT-CFD/FEM plus surface-to-surface radiation workflow can be justified. However, the case for this added complexity must show after accounting for measurement noise, parameter identifiability, and computational cost statistically and operationally meaningful improvements over simpler surrogate models.

#### 4.2.2. Empirical Models

Empirical models are often preferred when rapid, explainable temperature predictions are required under operational uncertainty, such as unknown ambient winds during roll-out or variable taxi profiles. One common approach is to use correlation or map-based heat transfer closures that provide phase-aware convection and enclosure-radiation estimates without the need to run computationally intensive CFD simulations. These typically rely on rotating disk and cavity-flow correlations adapted from established heat-transfer literature [102]. A second class of approaches employs lumped-parameter models (RC—thermal resistance and capacitance) that represent brake, hub, rim, and tire components as a small number of calibrated nodes to capture ‘soak-back’ behavior and cross-component heat flow [111]. A third method involves experiment-calibrated identification procedures that tune interfacial heat input, partition ratios, and effective coefficients to match measured temperature histories, enabling simplified models to reproduce observed transient behavior [108,110,113]. When grounded in validated convection correlations and standard radiation methodologies, these empirical approaches can provide operationally useful cooldown predictions and support MBE/MQTW assessments. However, their fidelity and scope remain inherently more limited than those achievable through full CFD/FEM simulations.

##### Correlation and Map Based Heat Transfer Closures

Empirical boundary-condition models supply the convection and radiation terms needed to run fast solvers without CFD. During roll-out/taxi, convection on exposed brake faces is prescribed using rotating-disk in cross-flow correlations, and ventilation within the hub is captured with rotor–stator cavity relations; together, these provide phase-aware h(r,θ,t) that track the sharp loss of cooling as speed decays [102,108,133]. For through-flow rotor–stator cavities (ventilated stacks), validated literature maps drawn from established experiments and benchmarked computational studies supply local and annulus-averaged heat-transfer distributions that can be tabulated directly for empirical use [108,133,134]. After gear retraction, the wheel well behaves as a radiative enclosure in weak flow and a compact gray/diffuse treatment with measured emissivities and geometry-consistent view factors (or a radiosity formulation) captures the dominant exchange between hot rings/plates and the rim/liners [110,111]. Although these closures are computationally trivial, they are not generic because they embed aircraft-specific geometry and duty-cycle assumptions, and small deviations in configuration or operating phase can shift h and radiative exchange appreciably. Hence, their speed advantage only holds if they are calibrated and used within clearly verified boundaries.

##### Lumped-Parameter (RC—Thermal Resistance and Capacitance) Wheel–Brake–Rim–Tire Networks

RC (RC—thermal resistance and capacitance) networks model the friction stack, back/pressure plates, torque path, rim, and tire gas as thermal capacities linked by effective conductances (conduction interfaces; correlation-based convection; enclosure radiation). The braking heat input often represented as a finite pulse shaped by moving-source physics enters at the disc, and the network predicts both early stack peaks and delayed rim/tire soak-back. Practical construction and calibration of such networks are documented in SAE work and follow-on studies that use CFD or experiments to estimate effective conductances where ventilation dominates [133,134]. Evidence from stationary, heated-disc tests provide separate estimates of convective and radiative heat-loss coefficients. These values can be inserted directly into the resistive-capacitive (RC) network links [135,136,137]. Complementary studies on ventilated or porous-core rotors show that vane geometry and core porosity materially change the effective convective heat-transfer coefficient h required by the network to match observed cooling behavior [138,139]. For selecting practical parameter ranges and safety margins, standard brake design references remain a useful guide [140]. RC networks are fast, but their reliability hinges on how the lumped conductances and heat input are identified; without sensitivity checks and phase-wise validation, fitted h and radiation terms can absorb contact, material, and ventilation effects, producing predictions that do not generalize.

##### Experiment-Calibrated Identification (Inverse/Lookup Tuning)

As several key inputs are uncertain in service, such as the time-varying interface heat flux, heat-partition factor, contact conductance, and the phase-dependent convection coefficient h(t), empirical models are typically calibrated using data from instrumented rigs or flight-like tests, including thermocouples placed in the stack or rim and supporting IR measurements [113]. To infer the unknown boundary heat-flux history from interior temperature measurements, inverse heat-conduction problem (IHCP) techniques are employed, including regularized least-squares procedures, filter-based estimators, and adjoint formulations [118,141]. These inferred fluxes are then used to tune effective conductances and heat-partition parameters so that the reduced-order model reproduces the measured cooling behavior under representative conditions [142,143].

Good practice incorporates explicit verification steps, sensitivity and identifiability analyses, and uncertainty propagation to ensure that calibrated models provide decision-relevant confidence bounds on quantities such as cool-down time, rim or tire peaks, and fuse-plug margins [144,145,146]. IHCP-calibrated empirical models can reproduce observed cool-down transients with good fidelity. However, their reliability depends critically on adequate sensor coverage, suitable regularization choices, and parameter identifiability. Without phase-specific validation, fitted conductances and partition factors may compensate for unmodeled physics, leading to biased or misleading predictions when operating conditions change.

#### 4.2.3. Hybrid Models

Hybrid models blend physical fidelity with computational efficiency by combining a compact physics representation of the wheel–brake–rim system with measurement-based calibration and reduced order surrogates. They preserve essential thermo-physical mechanisms, transient conduction in the carbon stack and torque path, phase-dependent convection during roll-out and taxi, and enclosure radiation after retraction while avoiding the high cost of repeatedly solving full CHT or FEM models. Early applications of empirical brake-thermal calibration illustrate the value of anchoring reduced-order physics to measurement data, while more recent research has shown that hybridization becomes particularly effective when high-fidelity simulations are used to derive low-dimensional approximations suitable for fast evaluation [113]. Together, these combined strategies allow hybrid models to maintain physical interpretability while achieving runtimes compatible with operational use or parametric what-if studies. The remainder of this subsection details how hybrid models are constructed in practice, beginning with inverse-calibrated physics, followed by projection-based reduced-order formulations, and concluding with the verification, validation, and uncertainty quantification steps required to ensure decision-ready outputs.

##### Inverse-Calibrated Physics Within a Compact Multi-Physics Closure

A practical hybrid workflow begins with a compact forward model comprising transient conduction in the discs, back-plates, torque path, and rim, closed by correlation-based convection and gray-enclosure radiation. Because several thermal inputs are uncertain in service, including the time-varying disc heat flux, heat-partition/contact conductance, and phase-dependent convection coefficient h(t), these quantities are identified from temperature measurements obtained from embedded thermocouples or infrared imaging. Inverse heat-conduction techniques, particularly regularized least-squares and filter/smoother formulations, have been shown to stabilize the ill-posed inversion and provide reliable reconstructions for multilayer systems resembling brake stacks [141,142]. When the inferred heat-flux and conductance histories are reintegrated into the forward model, the resulting responses reproduce disc-stack peaks, rim heating, and tire soak-back behavior with low computational cost [143].

Reliable calibration requires accurate treatment of enclosure radiation. Measured emissivities and geometry consistent view-factor models or an equivalent radiosity formulation prevent the inversion from mis-tuning conduction or convection parameters to compensate for omitted cavity effects [144,145]. When radiation closure is handled correctly and sensor placement is adequate, compact IHCP calibrated models reproduce measured transients effectively. Without phase-specific validation or appropriate regularization, however, fitted parameters may become non-physical and degrade under off-design operating conditions.

##### Projection-Based Reduced-Order Models Derived from CHT/FEM

Where high-fidelity CHT/FEM simulations are available spanning variations in vane geometry, inter-disc gap ratio, through-flow, and braking duty cycles, these temperature and heat-flux fields can be compressed into projection-based reduced-order models. Methods such as POD/Galerkin, GNAT, and space–time LSPG preserve the dominant thermal dynamics while reducing computational cost by orders of magnitude [144,145,146,147,148]. Their construction typically involves generating offline snapshots under representative roll-out, taxi, and wheel-well conditions, followed by identifying a reduced basis that captures key modal content associated with interface peaks, hot-spot motion, and soak-back timing.

Once trained, such ROMs can be driven online using aircraft-specific boundary profiles derived from accepted rotating-disk and rotor/stator/cavity correlations or through-flow maps, enabling phase-aware thermal prediction without re-solving the underlying flow [149,150]. The credibility of reduced-order models depends on the extent to which the training dataset covers the operational envelope, extrapolation to unseen ventilation, gap-ratio, or braking scenarios may displace hot spots or mistime thermal peaks. Consequently, error checking, sensitivity assessments, and off-design validation are required before using ROMs for operational assessments, cool-down predictions, or brake energy compliance checks.

##### Uncertainty, Verification/Validation (V&V), and Decision-Ready Outputs

Given their role in operational decision-making, hybrid models must quantify uncertainty and demonstrate clear verification and validation traceability. Recommended practice includes examining parameter uncertainty in the identified heat flux, partition factor, and convection coefficients and propagating these uncertainties to operationally relevant outputs, such as cool-down time, rim and tire peaks, and fuse-plug margins. Ensuring that convection and radiation closures remain within accepted correlation ranges and geometry-consistent enclosure models further strengthens model defensibility [151,152].

Established frameworks for uncertainty quantification and model validation covering Bayesian calibration, regularization diagnostics, and scientific-computing V&V provide structured approaches for generating defensible confidence bounds [153,154,155]. These frameworks are particularly useful when hybrid models are used outside their immediate calibration envelope. Diagnostic tools help identify over-regularized or non-identifiable parameter sets that may distort predictions under off-design conditions [156].

Radiation-sensitive phases, such as the wheel-well enclosure period, also require explicit checks to verify that view-factor formulations and emissivity models remain valid across the relevant geometries and temperatures. Broader uncertainty quantification (UQ) analyses further ensure that predictions remain conservative when aircraft-specific ventilation, gap ratio, or operational duty cycles deviate from those represented in the calibration dataset [157,158]. Ultimately, confidence in cooldown and rim/tire predictions depends on phase-aware validation of convection and radiation closures as well as on maintaining adherence to the model’s validity envelope, especially under operational variability [159].

## 5. Aircraft Wheel and Brake Temperature Prediction

Historically, the development of wheel and brake temperature monitoring has progressed through several distinct stages. Early approaches relied primarily on embedded point sensors and conservative empirical limits to support operational decision-making, providing limited insight into spatial thermal behavior. Subsequent advances introduced physics-based thermal modeling, allowing for a more improved understanding of heat-transfer mechanisms within the brake and wheel assembly. However, this is often at the cost of high modeling complexity and limited operational applicability. More recent research has explored data-driven and AI/ML based prediction methods, motivated by the availability of onboard sensor data and increased computational capability. However, these approaches have largely been constrained by sparse sensing and a lack of spatially resolved, real-flight thermal datasets. As a result, current state-of-the-art efforts increasingly focus on hybrid strategies that integrate operational sensing, heat-transfer physics, and intelligent prediction models, aiming to balance physical fidelity with practical deployability. This evolution shows a shift from isolated sensing or modeling solutions toward integrated frameworks capable of supporting condition monitoring and predictive decision-making under real operational constraints.

**Figure 9:**
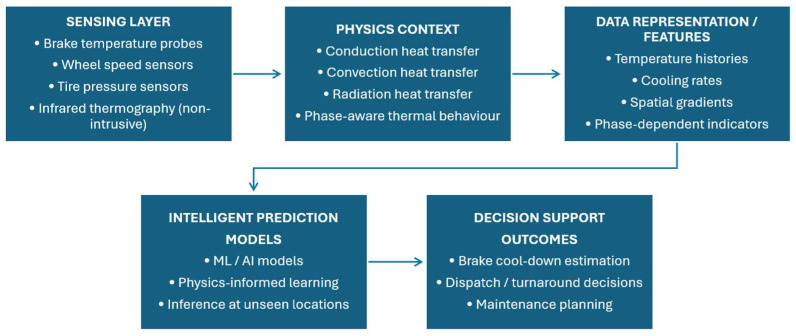
Conceptual integration of sensing, heat-transfer physics, and intelligent prediction models for aircraft wheel and brake temperature monitoring.

To clarify the functional interdependencies between sensing, thermal modeling, and prediction, Figure 9 presents a conceptual integration framework synthesized from the reviewed literature. Rather than proposing a specific implementation, the diagram illustrates the typical flow of information between sensing modalities, physics-based understanding of heat-transfer processes, and intelligent prediction models, highlighting how these elements collectively influence the accuracy and reliability of wheel and brake temperature prediction.

The preceding sections have examined the sensing landscape for landing gear systems, the capabilities and limitations of remote and embedded measurements, and the thermo-physical mechanisms governing wheel–brake behavior during landing, taxi, and retraction. Taken together, the literature highlights a growing need for reliable, scenario-aware temperature prediction to support operational decision-making, enhance safety margins, and reduce dependence on conservative cooling charts. Although extensive work exists on brake energy absorption, heat-transfer mechanisms, and numerical or empirical models, these studies are not tightly connected to operational data or to modern condition-monitoring practices. As a result, the ability to forecast brake temperatures under real conditions remains limited.

This section synthesizes the key insights from the literature to identify the dominant research gaps and emerging opportunities for advancing wheel and brake temperature prediction, followed by an assessment of the practical, scientific, and operational challenges that currently constrain progress.

### 5.1. Research Gaps and Opportunities

A review of the existing literature reveals that progress in wheel and brake temperature prediction is shaped by several interconnected limitations, each of which also presents opportunities for advancement.

The first major gap concerns the limited availability of real-flight thermal datasets. Most reported studies rely on laboratory dynamometer tests, controlled simulations, or simplified analytical approximations. These approaches provide valuable insight but do not capture the natural variability introduced by differences in landing mass, braking strategy, taxi duration, environmental conditions, and wheel-well ventilation. Generating systematically acquired, real-flight thermal data using non-intrusive methods is therefore a key opportunity for improving model fidelity and validation.

A second gap relates to the level of multi-physics integration in existing models. Conduction, convection, and radiation have each been studied extensively, yet few models combine these mechanisms across the full roll-out, taxi, retraction, and wheel-well phases. Critical transitions, such as the rapid shift from strong external convection during landing to enclosure-dominated cooling after retraction, are often oversimplified or omitted altogether. Addressing this gap offers the opportunity to develop phase-aware prediction models capable of capturing transient and spatially variable cooling behavior with greater accuracy.

Thirdly, the sensing practices also present both a limitation and an opportunity. Embedded sensors in current commercial aircraft applications are predominantly point-based and are not designed to characterize the distributed thermal state of the brake assembly. In contrast, infrared thermography can provide spatially resolved temperature fields without requiring aircraft modification. Despite its suitability for operational data acquisition, thermography has seen little application in published brake cooling studies. Leveraging this sensing modality creates a pathway for richer datasets, improved physics-based calibration, and more robust data-driven modeling.

A final gap lies in the application of advanced predictive modeling techniques. While machine learning approaches are now widely used across engines, APUs, avionics, and structural monitoring, comparable efforts for brake temperature prediction remain limited. No studies demonstrate the use of sequence-based learning, hybrid physics-aware models, or similar methods trained on operational thermal measurements. This represents a clear opportunity to exploit modern data-driven approaches, particularly when supported by improved sensing and systematically generated thermal datasets.

Overall, the literature points toward a promising direction combining non-intrusive thermal measurement, phase-aware multi-physics modeling, and modern data-driven methods to create a cohesive predictive framework for wheel and brake temperatures. However, several challenges remain before such a framework can be realized in practice.

### 5.2. Challenges

Several scientific, operational, and implementation challenges must be addressed before a deployable temperature prediction capability can be achieved. Thermographic measurements are sensitive to emissivity variation, reflections, sensor angle, and surface condition, requiring careful calibration and standardized acquisition procedures to ensure data reliability. Operational variability, including differences in runway conditions, braking strategy, environmental state, and thrust-reverser use, introduces significant dispersion into thermal signatures, complicating model generalization unless datasets are sufficiently wide-ranging.

Though infrared thermography offers a non-intrusive means of capturing spatially resolved surface temperature fields, its operational use in aircraft wheel and brake applications remains limited by fundamental physical and practical constraints. IR thermography provides only surface temperature information and cannot independently resolve internal conductive heat-transfer pathways or fully decouple conduction–convection interactions within the brake stack, wheel rim, and tire assembly. As a result, the scarcity of real-flight thermal datasets should not be viewed as an isolated data-availability issue but rather as a direct consequence of the historical reliance on predominantly embedded sensing architectures on commercial aircraft and the limited deployment of spatial sensing techniques under operational conditions.

The brake assembly itself exhibits strongly coupled thermal behavior, with anisotropic conduction in the carbon discs interacting with rapidly evolving convection regimes and radiative exchange in confined geometries. Capturing these interactions consistently across all operational phases remains a demanding requirement for physics-based and data-driven models alike. In addition, deploying predictive capability within airline maintenance and operational workflows involves further constraints such as certification considerations, data-sharing limitations, and the need for interpretable outputs that can be trusted by engineers and flight operations. Addressing these challenges is essential for transitioning from theoretical or experimental models to an operationally viable predictive framework.

## 6. Summary and Conclusions

This review has examined the multidisciplinary foundations underpinning aircraft wheel and brake temperature prediction, spanning landing gear operations, sensing technologies, thermal physics, modeling approaches, and intelligent prediction methods. Current research provides valuable insights into component-level behavior, heat transfer mechanisms, and sensor technologies, yet it remains fragmented and insufficient for real-world predictive temperature estimation. Four key research gaps were identified: (i) the absence of real-aircraft thermal datasets, (ii) insufficient multi-physics integration in existing models, (iii) limited application of infrared thermography, and (iv) the lack of ML/AI-based thermal prediction models trained on operational data.

These gaps present significant opportunities for innovation. Infrared thermography enables non-invasive, spatially rich temperature measurement across wheel and brake subcomponents. Combined with environmental sensing, it provides a unique dataset for developing data-driven and hybrid ML models capable of forecasting transient cooling profiles. Such models align with emerging IVHM and CBM paradigms, offering pathways to improve safety, minimize unscheduled maintenance, optimize turnaround operations, and enhance aircraft dispatch reliability.

However, realizing these benefits requires overcoming notable challenges, including thermographic measurement uncertainty, operational variability, thermal–mechanical coupling complexity, and deployment barriers within regulated aviation environments.

Overall, the synthesis of the literature underscores a compelling need and a clear research trajectory for developing a novel, experimentally validated, engineering-informed, machine-learning-driven wheel and brake temperature prediction framework. The resulting framework represents a significant step toward establishing a new generation of condition-monitoring capability for landing gear systems.

## 7. Future Directions

The research gaps, opportunities, and challenges identified in Section 5 highlight several key areas where further work can substantially advance temperature prediction for aircraft wheels and brakes. A primary requirement is the development of comprehensive operational datasets that capture the true thermophysical behavior of aircraft wheel and brake assemblies across varied operating conditions. Non-intrusive thermography provides a particularly valuable path forward, as it requires no aircraft modifications to capture temperature fields across wheel and brake assembly subcomponents immediately after landing. Such multilocation measurements will strengthen not only the fidelity of purely data-driven models but also hybrid approaches in which physical models and learned components are jointly calibrated using a representative in service behavior. In this context, the creation of open and standardized thermal datasets represents a critical enabler for future progress. Such datasets should adopt a multi-modal structure, combining wheel and brake sensors with non-intrusive infrared thermography to capture both localized and spatially resolved thermal behavior. Clear labeling of key operational phases, such as landing roll-out, taxi, and parked cooling, can support more informed modeling and interpretation. In addition, datasets should express confidence limits, with explicit consideration of emissivity variation, environmental conditions, and measurement limitations inherent to in-service data acquisition. Where feasible, appropriate anonymization and data abstraction would facilitate broader data sharing while respecting operational and data control constraints. The adoption of these principles would support more consistent validation, cross-study comparison, and the development of robust predictive frameworks focused in real operational behavior.

A second direction involves improved characterization of the coupled thermal processes governing wheel and brake temperature evolution. While existing work has examined conduction, ventilation-driven convection, radiation, and soak-back in isolation, future studies should focus on capturing their combined, phase-dependent influence in a unified framework. Real operation datasets will enable deeper mapping of energy flow from the carbon heat pack into neighboring components and facilitate a clearer understanding of how these processes shape the overall thermal response of the assembly. Such insights will support the development of temperature prediction models that reflect full system behavior rather than isolated component trends.

A third direction is the advancement of hybrid modeling strategies that combine compact physics-based representations with data-driven components. Hybrid models are well positioned to exploit the richer thermal datasets enabled by non-intrusive sensing, allowing for time-varying heat fluxes across wheels and brakes to be estimated more accurately from real aircraft behavior. Future research should explore fast, lightweight hybrid architectures capable of capturing system-wide dynamics, including heat transfer into the tire and generating interpretable outputs suitable for engineering validation. Verification, validation, and uncertainty quantification should form a core part of their development to ensure robustness across the operational envelope.

Finally, practical implementation pathways require further exploration. For temperature prediction tools to support IVHM and airline maintenance operations, they must integrate reliably with existing operational methods and digital systems, respect airline data-governance constraints, and deliver outputs that are interpretable by maintenance engineers. Future work should examine how prediction models can be adapted to varied environmental conditions, incorporated into turnaround workflows, and used to provide actionable temperature-based advisories. Such developments would support the transition from threshold-based monitoring to predictive capabilities that enhance safety margins, improve aircraft dispatch reliability, and provide a robust foundation for wheels and brakes condition monitoring.

## Figures and Tables

**Table 1:** ICF MRO spend forecast (Constant 2016 USD) by aircraft type [11].

Landing Gear				
**Aircraft Type**	**2016 USD**	**2021 USD**	**2026 USD**	**10 Year CAGR**
737	126,607,924	172,959,470	233,317,783	6.30%
757	12,941,871	15,153,349	8,807,196	−3.80%
767	39,279,625	22,680,664	16,844,494	−8.10%
777	106,106,000	110,074,169	118,474,149	1.10%
A320	164,749,970	216,958,652	286,786,845	5.70%
A330/A340	128,597,411	109,383,526	105,065,685	−2.00%
Others	142,382,401	199,848,250	268,191,157	6.50%
Wheels and Brakes				
**Aircraft Type**	**2016 USD**	**2021 USD**	**2026 USD**	**10 Year CAGR**
737	1,071,024,431	1,510,547,070	1,829,370,863	5.50%
757	68,348,554	42,214,873	18,018,807	−12.50%
767	141,171,422	98,624,382	66,543,386	−7.20%
777	496,112,887	493,336,094	376,295,720	−2.70%
A320	1,290,127,338	1,751,379,270	1,983,752,344	4.40%
A330/A340	375,824,342	391,104,170	350,689,507	−0.70%
Others	1,231,267,956	1,610,700,814	2,131,974,077	5.60%

**Table 2:** Comparison of FAA and EASA certification/standard landscape for W&B sensors.

Aspect	FAA (United States)	EASA (Europe)	Refs.
Environmental and EMI Qualification	RTCA DO-160G—defines test procedures for temperature, vibration, humidity, shock, and EMI.	EUROCAE ED-14G—equivalent to DO-160G; harmonized requirements for environmental/EMI testing.	[33,34,35]
System Development Process	SAE ARP4754A for system development; SAE ARP4761 for safety assessments (FHA, FMEA).	Same SAE ARP standards adopted; referenced in EASA AMC/GM guidance for CS-25 compliance.	[36,37]
System Safety and Compliance Guidance	FAA Advisory Circulars (ACs) provide acceptable means of compliance, e.g., AC 20-97B for tire maintenance.	AMC 25.1309 and related GM—defines system safety objectives and acceptable compliance methods for large airplanes.	[38,39]
Operational/Maintenance Requirements	AC 20-97B—tire inflation, inspection, maintenance, and operational practices.	EASA Part-26 AMC/GM—continued airworthiness and operational safety for landing gear/wheel/brake assemblies.	[40,41]

**Table 3:** Machine Learning approaches for prediction models in aerospace applications.

Model Family	Representative Methods	Typical Data Type	Key Advantages
Conventional Feature-Based Learners	Random Forests, Gradient-Boosting Machines, Support Vector Machines (SVMs)	Structured/tabular data (engineered features from operational context: phase of flight, mass, wind, runway condition)	Transparent, interpretable baselines; robust in safety-critical applications
Sequence Models	LSTM Networks, Temporal Convolutional Networks, Transformer-based Models	Time-series data (sensor logs, thermal histories, FC)	Capture temporal dependencies, hysteresis, and lagging effects; suited for multi-horizon forecasting
Image-Based Models	Convolutional Neural Networks (CNNs), Three-Dimensional CNNs (3D-CNNs)	Infrared thermograms, thermal image sequences	Learn spatial and temporal thermal patterns; detect anomalies; extract features beyond hand-crafted rules
Cross-Cutting Considerations	Uncertainty quantification (aleatoric/epistemic), calibration (e.g., conformal prediction), validation (blocked/rolling splits)	Applicable across all data types	Ensure predictive reliability, avoid optimistic bias, and provide confidence bounds

## Data Availability

No new data were created or analyzed in this study. Data sharing is not applicable to this paper.

## Abbreviations

The following abbreviations were used in this manuscript:

AC	Alternating Current
ACMS	Aircraft Condition Monitoring System
AE	Acoustic Emission
AI	Artificial Intelligence
AMC	Acceptable Means of Compliance
BSCU	Brake and Steering Control Unit
CAGR	Compound Annual Growth Rate
CBM	Condition-Based Maintenance
CFD	Computational Fluid Dynamics
CHT	Conjugate Heat-Transfer
CM	Condition Monitoring
CNN	Convolutional Neural Network
CS	Certification Specification
DC	Direct Current
DL	Deep Learning
EASA	European Union Aviation Safety Agency
EMI	Electromagnetic Interference
ETSOs	European Technical Standard Orders
FAA	Federal Aviation Administration
FBG	Fiber Bragg Grating
FC	Flight Cycles
FEM	Finite Element Models
GM	Guidance Material
IHCP	Inverse Heat-Conduction Problem
IR	Infrared Thermography
IVHM	Integrated Vehicle Health Management
LSTM	Long Short-Term Memory Network
MBE	Maximum Brake Energy
ML	Machine Learning
MQTW	Maximum Quick-Turn Weight
MRO	Maintenance, Repair, and Overhaul
NNs	Neural Networks
PHM	Prognostics and Health Management
ROMs	Reduced-Order Models
SVs	Shop Visits
TBMU	Tire And Brake Monitoring Unit
TPMS	Tire Pressure Monitoring System
TSOs	Technical Standard Orders
UT	Ultrasonic Testing
W&B	Wheels And Brakes
ε	Emissivity
